# Multimodal holotomography for volumetric monitoring of host–pathogen interactions

**DOI:** 10.1186/s12938-026-01577-8

**Published:** 2026-05-07

**Authors:** Ziyang Yu, Lei Jin, Mingyang Liang, Aaron Au, Thaisa Luup Kannen, Peter Serles, Tobin Filleter, Laurence Pelletier, Scott D. Gray-Owen, Christopher M. Yip

**Affiliations:** 1 Institute of Biomedical Engineering, University of Toronto, 164 College Street, Toronto, ON M5S 3G9 Canada; 2 Donnelly Centre for Cellular & Biomolecular Research, University of Toronto, 160 College Street, Toronto, ON M5S 3E1 Canada; 3 Department of Chemical Engineering & Applied Chemistry, University of Toronto, 200 College Street, Toronto, ON M5S 3E5 Canada; 4 Department of Biochemistry, University of Toronto, 1 King’s College Circle, Toronto, ON M5S 1A8 Canada; 5 Lunenfeld-Tanenbaum Research Institute, Mount Sinai Hospital, Toronto, ON M5G 1X5 Canada; 6 Department of Mechanical and Industrial Engineering, University of Toronto, 5 King’s College Road, Toronto, ON M5S 3G8 Canada; 7 Department of Molecular Genetics, University of Toronto, 1 King’s College Circle, Toronto, ON M5S 1A8 Canada

**Keywords:** Holotomography, Quantitative phase imaging, Bacterium tracking, CEACAM, *Neisseria gonorrhoeae*, Host–pathogen interaction, Correlative microscopy, HILO fluorescence, HomoFRET

## Abstract

**Background:**

A strategic way to gain a deeper understanding of how infection occurs is to characterize the mechanisms of pathogen binding and uptake. To accomplish this, we have focused our efforts on developing approaches that enable real-time monitoring of the initial stages of pathogen binding and the dynamics of the corresponding cellular response, including how membrane receptors are recruited to the binding sites. Past efforts have relied largely on widefield fluorescence imaging to track receptor recruitment and pathogen uptake.

**Methods:**

In this work, we have applied holotomography (HT) to probe pathogen binding using 3D refractive index (RI) reconstruction and spatial–temporal bacteria tracking. While promising, we also report on the challenges encountered in developing a multimodal correlative HT-fluorescence approach for real-time visualization of pathogen uptake and engagement.

**Results:**

We report here on an imaging platform that integrates HT with multicolor fluorescence microscopy. The system can reconstruct quantitative RI volumes and perform single particle and pathogen tracking. We applied this system to monitor the interaction of *Neisseria gonorrhoeae* bacteria with live CEACAM-expressing HeLa cells, coupling HT with simultaneous two-channel highly inclined laminated optical sheet (HILO) fluorescence imaging to track the dynamics and spatial distribution of membrane receptors and bacteria within the same field of view. Simultaneous acquisition and co-registration of the phase and fluorescence channels resulted in a multicolor, temporally synchronized dataset of pathogen binding and receptor engagement, portending video-rate three-dimensional tracking of pathogen binding and uptake.

**Conclusions:**

The coupled platform we have developed has enabled direct volumetric imaging of the dynamics of pathogen binding and concomitant recruitment of membrane receptors on live cells. The platform closes a critical gap between optically sectioned fluorescence and label-free 3D phase imaging and can be adapted to other receptor–ligand systems that would benefit from correlated structural and functional insights.

**Supplementary Information:**

The online version contains supplementary material available at 10.1186/s12938-026-01577-8.

## Background

The cellular response to infection is a dynamic process that involves engagement of host cell surface receptors by the pathogen’s adhesin molecules. This then triggers intracellular signaling events that may lead to inflammation, pathogenesis and/or pathogen engulfment. Characterizing on relevant spatial and temporal length scales how these processes, both protective and pathogenic, take place is therefore key to creating new opportunities for the development of therapeutics that either impair or enhance these mechanisms. Visualizing how pathogens engage the host cell, how receptors are recruited to the binding sites, their association states and the dynamics of these processes is therefore of particular interest. The carcinoembryonic antigen-related cell-adhesion molecule (CEACAM) family of glycoproteins are exploited by *Neisseria gonorrhoeae*, the pathogen responsible for gonorrhea, by the bacterial Opa protein adhesins binding to various human CEACAM receptors [1–3]. On the cell surface, the evolutionary progenitor of this family, CEACAM1, is known to exist in a complex self-association state comprising monomers, as well as homo-, and hetero-oligomers, but the exchange dynamics and implications for infection remain in question. While Opa binding to human CEACAM1, CEACAM3, CEACAM5 and/or CEACAM6 allows bacterial entry into the host cell, the mechanism of bacterial engulfment differs depending upon which CEACAM is engaged. For example, CEACAM3 mediates an efficient phagocytosis-like engulfment of the bound bacteria through a process that activates the cell [4], while CEACAM1 engagement inhibits cellular responses and induces a process resembling endocytosis [2, 5, 6]. Real-time observation of pathogen binding and concomitant CEACAM receptor recruitment dynamics at the host cell membrane is therefore essential to understand the mechanisms of infection, particularly given that multiple CEACAMs are expressed on many cell types.

Obtaining real-time insights into CEACAM–*N. gonorrhoeae* interactions represents a significant imaging challenge with a set of conflicting design requirements [7]: (1) resolving pathogen trajectories before attachment to the host cell; (2) identifying binding events, and (3) monitoring of the host cell membrane structure and dynamics, ideally all over the same field of view. For example, spinning-disk confocal microscopy, which is a powerful tool for studying cellular dynamics and structures, is not well suited for simultaneously tracking individual pathogens, especially if they are transiting to or from the host cell surface [8, 9]. While techniques such as highly inclined laminated optical sheet (HILO) microscopy provide axially sectioned widefield fluorescence appropriate for tracking both receptor recruitment and pathogen binding [10, 11], it is not well suited for dynamic 3D imaging and particle localization due to the limitations of stage scanning. Of note, the aforementioned techniques are all fluorescence-based approaches, which can be problematic, depending on the availability and appropriateness of a given fluorophore. We would further note that 3D tracking of pathogen movement and dynamics by fluorescence would require the pathogen to be appropriately labeled. 

Digital holographic microscopy (DHM) provides dynamic, label‑free quantitative phase imaging (QPI) [12] of single cell dynamics by interferometry [13, 14]. The DHM phase map is a topographical measurement of optical path length differences (OPD), which is the integral of refractive index (RI) contrast over the specimen thickness along the transillumination wave propagation axis. Because OPD conflates RI and axial thickness, DHM alone cannot distinguish whether a given phase change arises from a change in morphology or in specimen composition [15]. Holotomography (HT), a synthetic‑aperture extension of DHM, addresses these limitations by reconstructing a true three‑dimensional (3D) RI distribution from multi-shot QPI data. Leveraging off‑axis interferometry, which reliably retrieves OPD with ~ 100 nm sensitivity [16], coherent-illumination HT can resolve intrinsic 3D structural and compositional information at the single‑cell level [17, 18]. Operating in the in-line regime, DHM additionally facilitates 3D tracking of highly scattering particles from a single-shot image by utilizing numerical wavefront reconstruction, precluding the need for fluorescent labeling or hardware scanning as would be found in conventional multi-particle tracking instruments [19–21]. The recorded hologram can be numerically backpropagated to arbitrary axial distances a posteriori using various approaches such as the Fresnel, Rayleigh–Sommerfeld, or angular spectrum models [22]. In practice, the focal plane is identified by sweeping the reconstruction distance, assigning a focus score to each slice, and then selecting the maximum based on deterministic criteria [20, 23] or machine learning [24].

As a label-free technique, HT can only provide morphological details and therefore lacks chemical or molecular specificity. One way to address this would be to develop a correlative multimodal approach that combines the phase information with fluorescence imaging [25, 26]. To date, most multimodal HT systems are configured with widefield epifluorescence [26–31] and therefore suffer from limited surface specificity and sub-optimal signal-to-noise ratios due to background fluorescence (Supplementary Table 1). While HILO’s oblique illumination [10] improves sample axial sectioning, it is susceptible to interference fringes [32] as well as intensity profiles skewed toward the excitation spot, complicating direct quantitative analysis of the intensity [33]; several optical designs have been developed to homogenize the HILO illumination profile through azimuthal steering of the laser beam [32, 34]. Lastly, most current multimodal HT implementations either rely on specialized components such as wavefront sensors [30], which would preclude truly simultaneous HT and fluorescence acquisition [26, 27], or restrict imaging to a single fluorescence channel [29].

To overcome these limitations, we report here the development of a multimodal holotomography system that allows for both single-shot spatial-volumetric pathogen tracking as well as 3D refractive index imaging. This system is also the first correlated HT platform that integrates azimuth-scanned HILO fluorescence excitation and simultaneous multichannel detection. This integration enables simultaneous acquisition of 3D RI contrast or quantitative localization and two-channel volumetric fluorescence data. We applied this approach to image HeLa–*N. gonorrhoeae* interactions with dual-color labeling, and fluorescence anisotropy homo-Förster resonance energy transfer (HomoFRET) measurement of live cell CEACAM1 [35] recruitment, demonstrating its potential for more detailed studies of infection.

## Results

### Holography and holotomography imaging of step targets

**Fig. 1:**
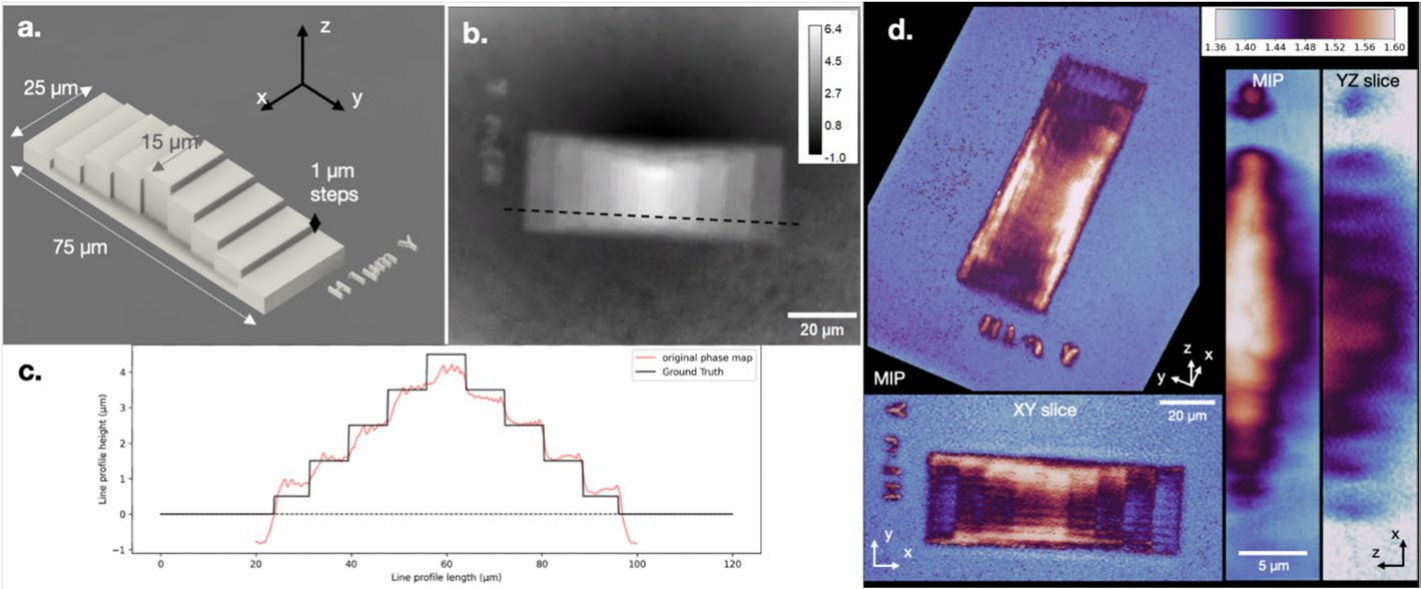
Extraction of phase and RI tomogram from the step target. **a** Step target design parameters. **b** Height map of the step target, all taken from the 60th image of the galvo scanning process, where the galvo is centered in scanning position. **c** Cross-section profile corresponding to the section in **b**. **d** Tomographic reconstruction of the step target, showing both the maximum intensity projection (MIP) rendering as well as a sliced section of the target (X–Y slice at base of target, Y–Z slice at the center of the target). Y-axis scale bars: 20 µm; z-axis scale bar: 5 µm

The HT system was first evaluated using a step target patterned on a glass slide (Fig. 1). For each single-shot multiplexed off-axis hologram, Fourier-domain bandpass filtering isolated the carrier sidebands, yielding complex fields at 660 nm. The phase data show good agreement between the real-space target step dimension and the measured phase data. Subsequently, the RI tomogram was generated from the angle-multiplexed phase data. Despite the refractive index difference between the immersion medium and the adverse effect of the missing cone artifact from a limited numerical aperture, the reconstruction exhibits accurate ridge boundaries (Fig. 1d). (We have provided an extended assessment of the spatial resolution, RI accuracy, and sample–medium mismatch in Supplementary information 2 and 3.) These results establish that the HT subsystem accurately retrieves both phase-derived height and volumetric refractive-index structure.

### Fluorescence and holographic imaging of nano-printed phantoms

**Fig. 2:**
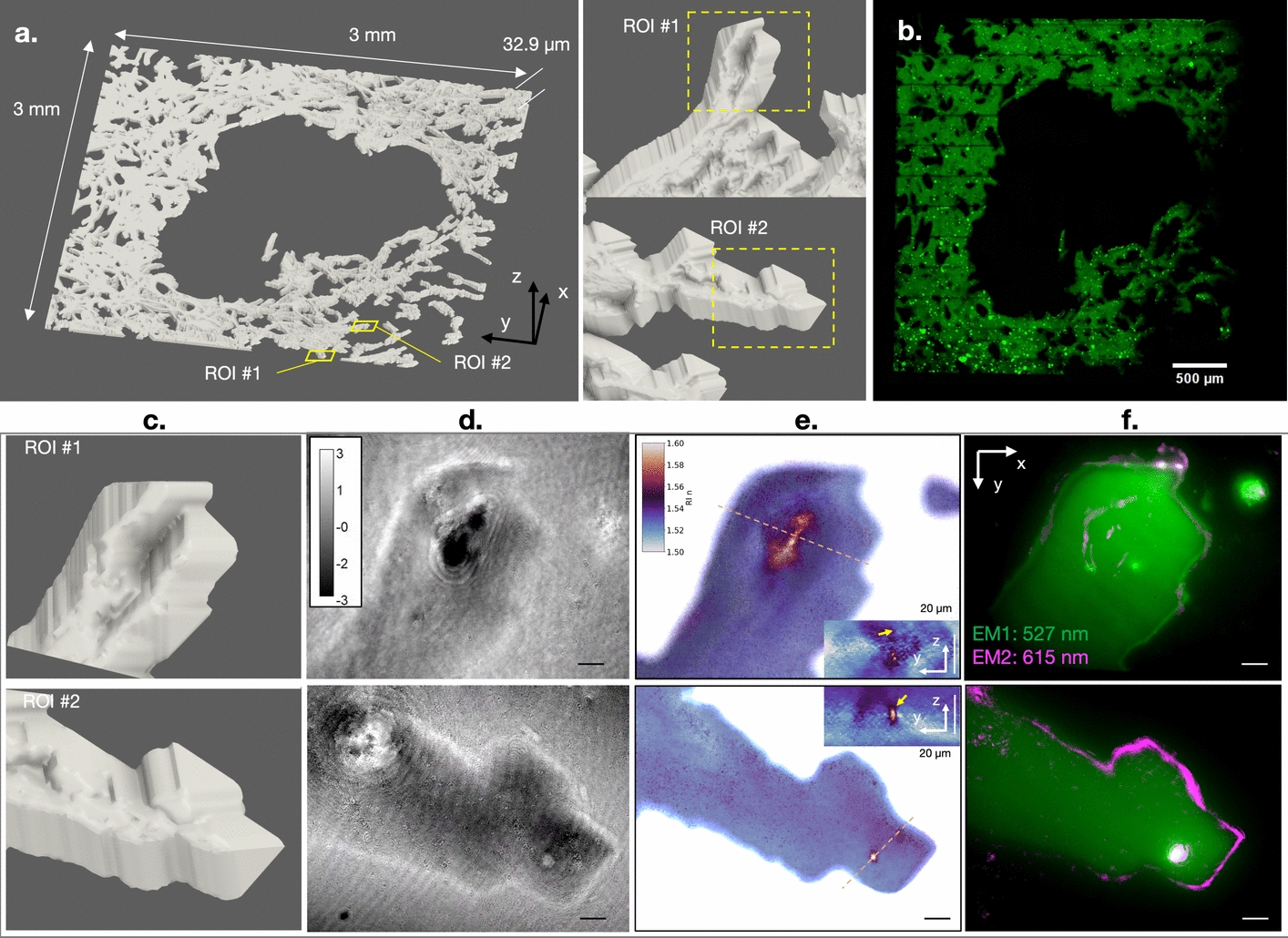
Correlative phase, RI and fluorescence imaging of the synthetic vascularized organoid phantom. **a** 3D rendering and specifications of the organoid phantom, where two regions of interest (ROI) are acquired on the HT system using 60 × magnification. **b** Stitched scanning laser confocal maximum intensity projection (MIP) image of the full vascularized organoid. **c** The two ROIs magnified. **d** Individually unwrapped phase image on the two ROIs. **e** An MIP of RI tomograms of the two regions at the image plane. Top inset: axial-section RI slice of the hollow lumen, indicated by the arrow. Bottom inset: axial-section RI slice of the vertical profile drawn over the bead puncta. **f** Registered and layered HT and volumetric HILO fluorescence image (527 nm and 615 nm emission peaks) of the two ROIs. All scale bars are 10 µm, except those of the insets

The nano-printed organoid phantom, a biologically inspired fluorescent specimen with weak intrinsic contrast and a thick height profile, was used to validate the colocalization of the two modalities within the system (a description of the design and fabrication process is provided in the Methods section). Two ROIs were acquired at 60× magnification on the HT system **(**Fig. 2a**, **Fig. 2c**)**, with ROI-1 containing a tapered lumen and ROI-2 spanning a vessel-like wall. Both ROIs contain fluorescent beads at various axial localizations.

For each ROI, we reconstructed the complex fields and performed phase unwrapping (Fig. 2d). The result exhibits consistent path-length topographies that delineate wall boundaries and lumen interiors despite the small polymer–oil index contrast. Local phase gradients are highest at the wall edges and around bead locations, as expected from the large RI difference of the polystyrene spheres. Angle-resolved datasets were then inverted to refractive-index tomograms (Fig. 2e). The lumen geometry in ROI-1 was visualized with slightly lower RI, at *n* ≈1.51, than the rest of the phantom with RI values in the range of 1.52–1.54. The polystyrene bead morphology in ROI-2 can be clearly recovered (Fig. 2e inset), where an RI peak of *n* ≈ 1.586 can be observed.

Finally, we registered the RI tomograms with volumetric HILO fluorescence (Fig. 2f). The green channel outlines the nanoparticle-labeled walls, whereas the red fluorescence channel localizes the 1-µm beads. A one-to-one correspondence between high-RI ridges and green fluorescence indicates structural co-registration. They also show that the red fluorescent beads coincide with high-RI puncta on the surface. Together, these results demonstrate that multimodal HT can recover volumetric refractive-index structure in a weak contrast, biologically relevant phantom, and deliver quantitatively co-registered structural (RI) and functional (fluorescence) information suitable for downstream correlative analyses. The resulting RI volumes recover the hollow lumen wall thickness variations. The RI values of the near-wall voxel cluster and surrounding medium were *n* ≈ 1.52 and 1.51, respectively.

### Correlative quantitative phase–single-shot HILO and RI–volumetric HILO imaging of CEACAM1 in HeLa cells and N.* gonorrhoeae*

**Fig. 3:**
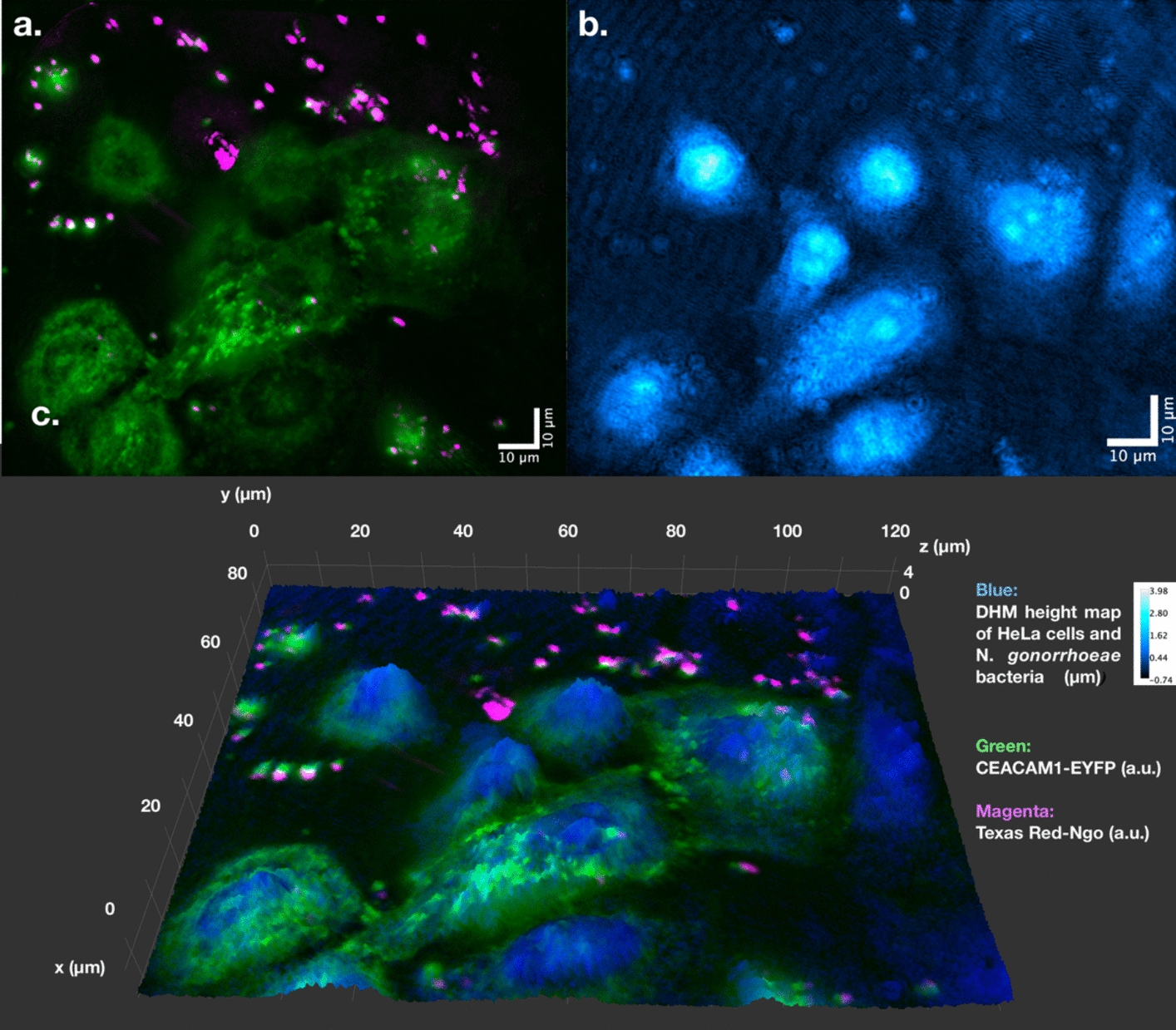
Correlative HILO fluorescence and DHM image of *N. gonorrhoeae* on and around live HeLa cells. **a** Widefield HILO fluorescence image, two-color CEACAM1-EYFP expressing HeLa and Texas red-labeled *N. gonorrhoeae*. Texas Red-Ngo, Texas Red-*N. gonorrhoeae*. **b** DHM height plot, calibration bar unit is in µm for sample height. **c** Co-registered HILO and DHM 3D height plot rendering. Blue: DHM quantitative height map

**Fig. 4:**
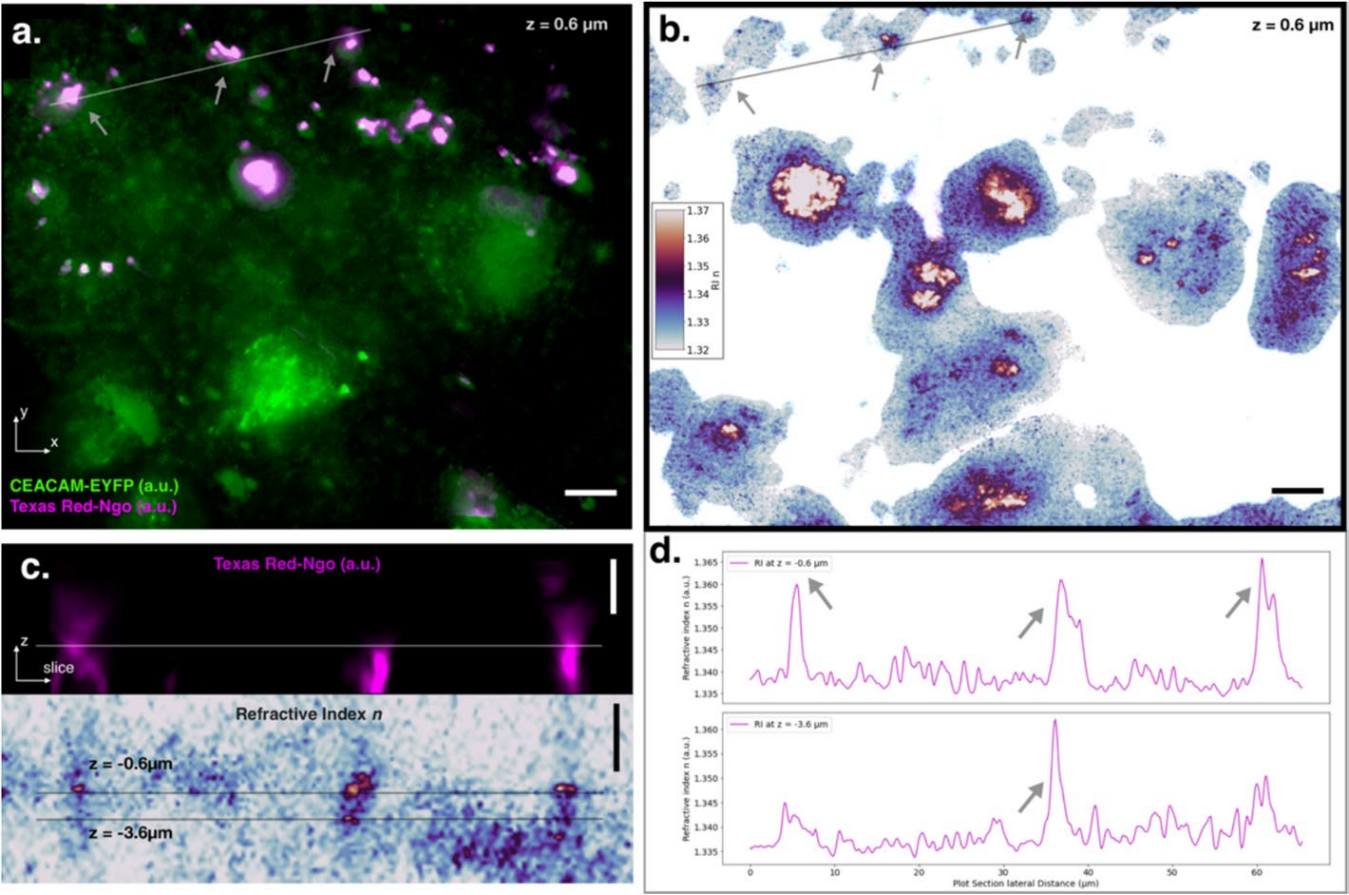
RI tomogram of inactivated *N. gonorrhoeae* on and around fixed HeLa cells. **a** Two-color, axially sectioned HILO under the same field of view in **(**Fig. 3**)**. **b** Correlative RI at the same focus plane. **c** Axial slice image from RI tomogram and HILO volume at two different z steps, measured from the sections in **a** and **b**. Texas Red-Ngo, Texas Red–*N. gonorrhoeae*. **d** Line plot profile measuring *N. gonorrhoea*e bacteria RI at two axial reconstruction depths taken in **c**

We assessed the multimodal capability of the system on live HeLa cell monolayers expressing CEACAM1-EYFP after *N. gonorrhoeae* exposure. HILO data were co-registered to the simultaneously acquired DHM phase-to-height map (Fig. 3) and RI tomogram **(**Fig. 4), respectively. Comparison of the widefield fluorescence and QPI revealed that DHM can resolve sub-cellular structures such as the nucleus of the HeLa cells, as well as sites of individual bacteria. These images also revealed that a few points of bacterial attachment, located at the cell membrane, were encircled by green CEACAM1 enrichment. Some of the bacteria were also found not to be associated with the cells, lying in regions of exposed glass.

Orthogonal slices through the 3D stack show that CEACAM1 accumulation occurs at the membrane–bacterium interface rather than uniformly across the cell surface, and that receptor-rich patches track the local membrane topography measured by DHM (Fig. 4). HILO sectioning reduces out-of-focus background relative to widefield epifluorescence, allowing bacteria adjacent to steep membrane features to be resolved and assigned to the correct axial plane.

Figure 4c further showcases two registered slices from the refractive-index (RI) tomogram overlaid with the volumetric HILO stack of the Texas Red channel, separated by an axial offset of 3 µm (top: *z* = 0.6 µm; bottom: *z* = 3.6 µm). In the HeLa cells, one can observe the RI value of the nucleus to be lower than that of the surrounding cytoplasm, while islands of high-RI regions can also be observed in the nucleus. At *z* = 0.6 µm, many fluorescent puncta coincide with RI hotspots located on or immediately above the apical membrane, whereas others lie off-cell over regions of near-uniform, and with lower RI. Shifting the plane to *z* = 3.6 µm attenuates several of the on-membrane peaks and reveals different puncta with stronger RI contrast, consistent with bacteria residing at distinct axial positions relative to the surface. N. *gonorrhoeae* produces a high RI contrast on the epithelial cell layer at* n* ≈ 1.37, and the lateral widths are on the order of a micron, matching the bacterial size (Fig. 4b). RI tomography and HILO fluorescence provide complementary readouts in the same field of view, where fluorescence marks bacterial identity, while the RI map supplies label-free structural context and axial positioning.

### Correlative single-shot 3D *N. gonorrhoeae* tracking alongside HILO live cell imaging

**Fig. 5:**
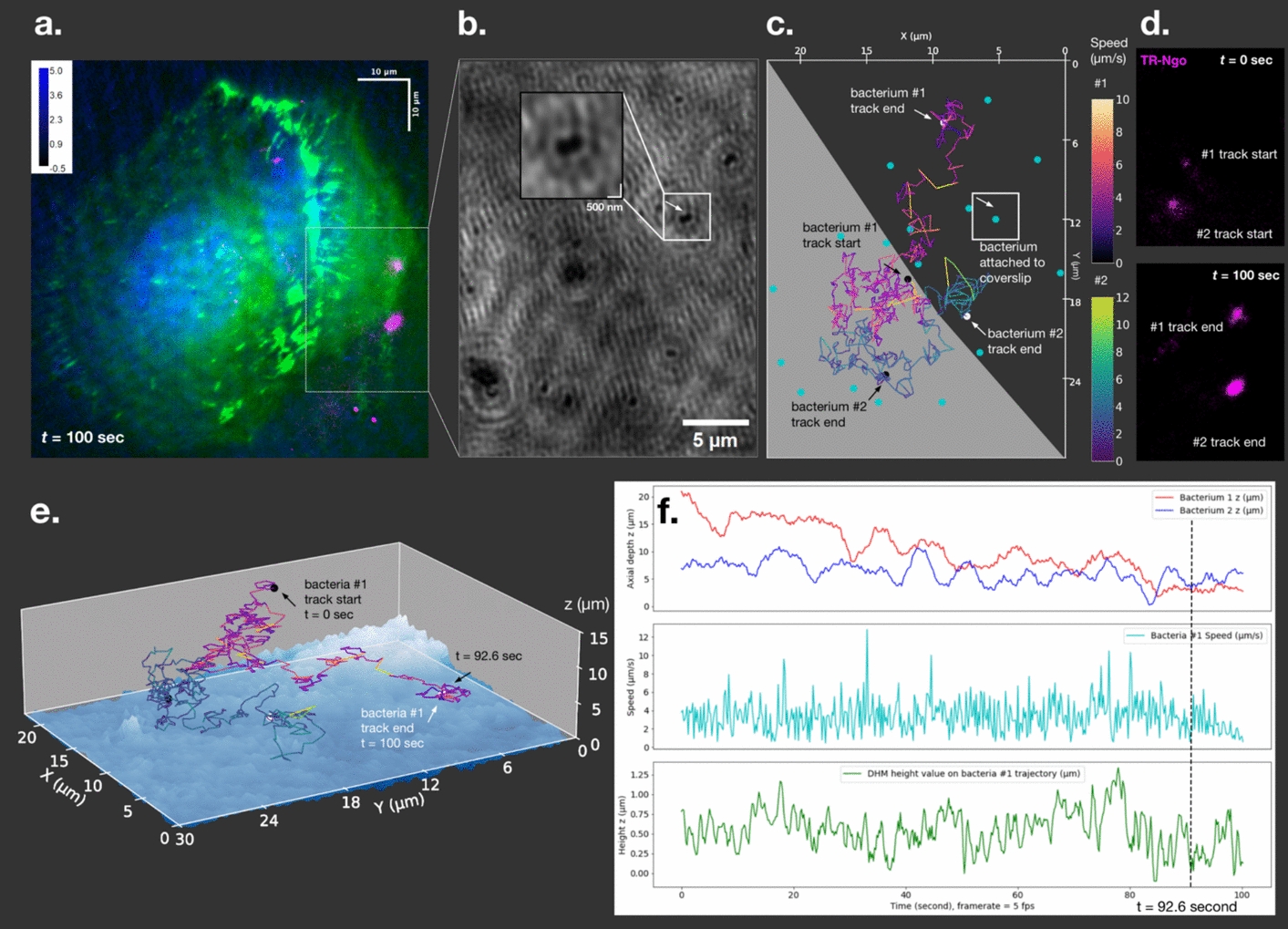
Correlative tracking of *N. gonorrhoeae* and HILO during live imaging. **a** Correlative widefield DHM-HILO image at the end of the time-lapse. Green: CEACAM1-EYFP; magenta: *N. gonorrhoeae* (Texas Red); blue and scale bar: DHM height map (µm). **b** A numerically refocused DHM ROI containing the immobile bacteria on the coverslip. Inset: *N. gonorrhoeae* bacterium. **c** Two bacterial trajectories from the same ROI as **b**, color-mapped with bacterial movement speed, plotted alongside immobile bacteria. **d** Texas Red–*N. gonorrhoeae* validation at the start and end of the time-lapse. TR-Ngo, Texas Red–*N. gonorrhoeae*. **e** The two bacterial trajectories in 3D, plotted against the DHM phase-to-height surface map. **f** *N. gonorrhoea*e bacteria trajectory axial position, speed, and DHM height values along the trajectory of the time-lapse

Next, we used the spatial–temporal tracking technique (Fig. 5) to correlate *N. gonorrhoeae* movement with HILO fluorescence in vivo. To confirm the size and morphology of the inactivated bacterium, holograms recorded with the stage set ~6 µm above the coverslip were numerically backpropagated to the focal plane. Based upon this, the major axis length of the bacteria is $0.854 \pm 0.077 \upmu \mathrm{m}$ (mean, standard deviation). Bacteria adhered to the glass were identified by applying a pixel-invariant temporal median filter to the hologram sequence prior to propagation, yielding a static background map used to exclude non-motile puncta (Fig. 5b).

Time-lapse imaging of *N. gonorrhoeae* commenced 5 min after perfusion and continues for 30 min. In a 100-s imaging window, multimodal HT enabled the tracking of two motile bacteria diffusing from the top of the sample dish, tumbling along the cell contour, and ultimately stopping at the edge of the epithelial surface. As the bacterial trajectories were linked over the time-lapse, instantaneous speed was computed from inter-frame Euclidean displacement. Two representative tracks are shown in Fig. 5c: bacterium-2 exhibited Brownian-like motion at an approximately constant axial height; in contrast, bacterium-1 descended toward the epithelial surface and decelerated near the end of the track. We set the z-stage 6 µm away from the coverslip throughout the time-lapse, allowing time-series HILO fluorescence of Texas Red–*N. gonorrhoeae* to be recorded in parallel with DHM data. The start and end positions of the DHM-derived tracks are colocalized with HILO (Fig. 5d), providing an independent lateral report for the particle identity.

Multimodal analysis of axial position, speed, and local DHM height along bacterium-1’s path (Fig. 5f) revealed a coordinated deceleration event during the time-lapse. At 92.6 s, speed dropped to 0.564 μm per second from a pre-event mean of 3.104 μm per second, coincident with a slight increase in axial position, a 0.21 μm decrease in the underlying DHM height from a localized negative gradient on the epithelial surface, and a change in lateral motion direction. These features are consistent with contact and stabilization at a membrane ridge [36, 37].

Together, the results demonstrate that correlative DHM–HILO provides synchronized structural and functional readouts to recover full 3D trajectories with per-frame speeds at the camera rate, and is able to isolate putative landing events where bacterial slowing coincides with membrane topography changes.

### Correlative HT, multicolor HILO, and fluorescence anisotropy imaging of CEACAM1 in vivo recruitment activities

**Fig. 6:**
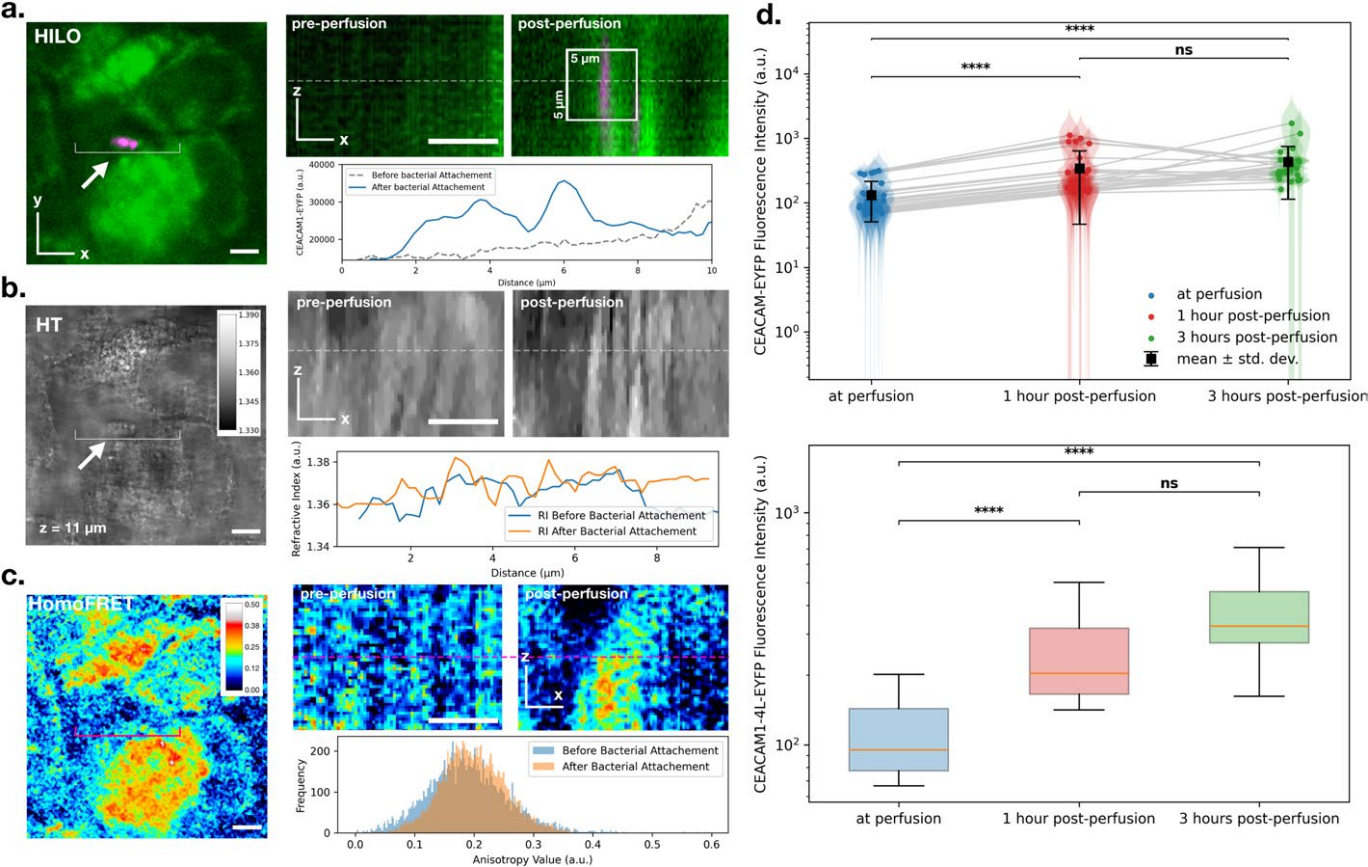
Correlative HILO-HT-HomoFRET imaging reveals CEACAM1 recruitment activities upon *N. gonorrhoeae* binding. **a** Volumetric HILO image at perfusion and 1 h post-perfusion; inset figure: line section plot of CEACAM1-EYFP intensity (green) and *N. gonorrhoeae* (magenta, Texas Red) localization. **b** HT RI tomogram (*en face* figure at z localization of 11 µm); inset figure: RI profile along the line showing morphological changes during bacterial engulfment. **c** HomoFRET anisotropy map of CEACAM1-4L-EYFP; inset figure: anisotropy distribution of the two X–Z sections taken before and after bacterial perfusion. **d** Quantitative measurement of CEACAM1-4L-EYFP intensity at bacterial attachment sites (n = 61 total) before and after perfusion. Each violin distribution at every time point represents the quantification of a $$125 {\upmu \mathrm{m}}^{3}$$ volume around the bacterial morphological mass center. Statistical significance ($$****\mathrm{P}<0.0001$$) was determined via paired-t and Wilcoxon tests. In all **a b c** inset: top left: X–Z section before perfusion; top right: X–Z section imaged 1 h after perfusion. Main figure scale bars: 20 µm; inset scale bars: 5 µm

To investigate the spatiotemporal dynamics of CEACAM1 during *N. gonorrhoeae* infection, we utilized multimodal HT for simultaneous multichannel imaging of the recruitment response. Prior to bacterial perfusion, baseline HILO, HT, and HomoFRET measurements were performed from the basal cell surface to a height of 15 µm (Fig. 6a–c). Under these resting conditions, CEACAM1-4L-EYFP exhibited a heterogeneous distribution characterized by a mixture of diffuse low-density regions and accumulated high-density clusters. Anisotropy distribution analysis revealed that CEACAM1 preferentially exists in a monomeric state within low-density regions, while shifting toward dimeric or oligomeric forms within high-density clusters, consistent with previous reports [35]. Volumetric HILO stacks further demonstrated axially dependent heterogeneity in CEACAM1 distribution (Fig. 6c). HT imaging of live cells captured dynamically forming filopodia and intercellular stress fibers, which exhibited a higher refractive index (RI) relative to the surrounding medium and nucleoli.

Following the perfusion of *N. gonorrhoeae*, HT imaging was employed to monitor bacterial attachment and subsequent membrane activities. As HT provides a consistent structural morphology of the bacterium free from tissue-induced fluorescence PSF distortion, we derived bacteria localizations from thresholding the RI tomogram and assessed 1 $25 {\upmu \mathrm{m}}^{3}$ area blocks around each bacterial attachment for CEACAM1-EYFP intensity and mean anisotropy values. Out of 61 identified bacterial attachment sites, 7 instances of definitive bacterial uptake were observed within the imaging window (Fig. 6b). Upon attachment, an enrichment of CEACAM1 was detected at the bacteria–cell interface. Quantitative analysis of the attachment sites revealed a significant increase in CEACAM1-EYFP fluorescence intensity from the time of perfusion to 1-h post-infection (****P < 0.0001, Fig. 6d). From 1 to 3 h post-perfusion, we observed 5 additional bacterial attachment events, and decreased variance of the accumulation of CEACAM1-EYFP intensities around previously attached bacteria. Correlative RI section plots (Fig. 6a, b) confirmed that bacterial engulfment by HeLa cells coincided with a localized increase in both CEACAM1 concentration and RI values. An increase in anisotropy values implies that the recruited CEACAM1 at these apical sites underwent a shift to higher monomer concentration during the transition from attachment to internalization. Collectively, these data demonstrate that HT-HILO-HomoFRET imaging can successfully resolve the mechanical and molecular rearrangements, specifically the recruitment and oligomerization of CEACAM1, that facilitate *N. gonorrhoeae* entry into host cells.

## Discussion

We have shown that integrating holotomography (HT) with volumetric fluorescence microscopy offers a powerful platform for characterizing structural events and dynamic processes at cell surfaces. In the case of CEACAM1–*N. gonorrhoeae* interactions, this approach is further strengthened by 3D bacterial tracking, made possible through single-shot DHM’s capacity for axial numerical reconstruction. The ability to conduct label-free, multi-bacterium tracking in a single exposure enables rapid monitoring of bacterial run–tumble dynamics without the need for mechanical scanning or stage movement, thereby minimizing sample perturbation; correlating DHM height maps with particle localizations enables the label-free tracking of pathogen attachment relative to epithelial topography**.** Moreover, fluorescence channels that would otherwise be dedicated to bacterial detection are freed for co-expression analysis or anisotropy studies. We have also demonstrated the feasibility of performing correlative RI imaging and azimuth-scanned illumination HILO live cell microscopy. HT provided the label-free structural context by mapping the cell membrane and surface topography, while HILO microscopy enables localization of receptors and pathogen binding. This fusion allowed us to directly observe receptor recruitment to bacterial contact sites. We validated the correlation of modalities through both in-house fabricated organoid phantoms, fixed samples, and live cell assays.

We have additionally improved the current arts in multimodal HT on two aspects: azimuth-scanning HILO excitation enables precise displacement of the excitation angle, which resulted in an expanded imaging field of view, improved sample illumination homogeneity, and reduced laser speckle noise. Multiple simultaneous detection channels offer unique advantages such as the ability to operate the detectors at different exposure times and frame rates for pathogen tracking or recruitment measurements, compatibility with quantitative fluorescence anisotropy methods, and allow for a larger effective imaging area than optical detection splitting. Indeed, we demonstrated the system’s applicability for quantitatively assessing recruitment of CEACAM1 in multimodal live imaging and identified a statistically significant enrichment around attached or engulfed pathogens. As CEACAM enrichment analysis was performed by retroactively localizing the pathogen for cross-checking fluorescence intensity, we envision the development of a reactive imaging pipeline where the tracking states of the pathogen actively trigger fluorescence characterization of the binding sites for membrane receptor assays.

While we have shown that the quantitative accuracy of the HT platform using engineered step targets and phantoms with known refractive indices, HT is still subject to the inherent limitations of angle-scanned tomography, namely the axial missing frequency artifact during reconstruction. For real-time multimodal assessment of the host–pathogen dynamics, challenges remain in further improving detection throughput of both HT and fluorescence to allow video-rate tracking of dynamics. Recently, digital micromirror (DMD) based HT has been successfully demonstrated to achieve 1-kHz measurement for sparse bacterial pathogens [38], and at video rate with a non-interferometric design with angular multiplexing [39]. These works utilized advanced diffraction propagation models for HT reconstruction to reduce the missing cone artifact and the number of acquisition shots required for a tomogram [40]. However, the imaging SNR at a high multiplexing degree is not sufficient for resolving sub-cellular details of cellular cultures. On the spectrum of fluorescence microscopy, excitation beam scanning [41, 42] has witnessed adoption over sample scanning for high-throughput cell and tissue imaging. The marriage of the aforementioned technologies could signal a feasible path toward implementing a real-time multimodal HT for studying live cell host–pathogen dynamics.

## Conclusion

In summary, we have developed and validated a multimodal imaging platform that provides co-registered structural and fluorescence information with high quantitative resolution. The methodology is well suited for investigating dynamic events at the cell–environment interface, including receptor–ligand interactions, pathogen engagement, and membrane remodeling. With further optimization for acquisition speed and the expansion to additional fluorescence channels, this platform is poised to enable routine spatial–temporal studies of host–pathogen interactions and other complex membrane-associated processes, thereby facilitating more mechanistic, hypothesis-driven investigations.

## Methods

### Experimental setup

**Fig. 7:**
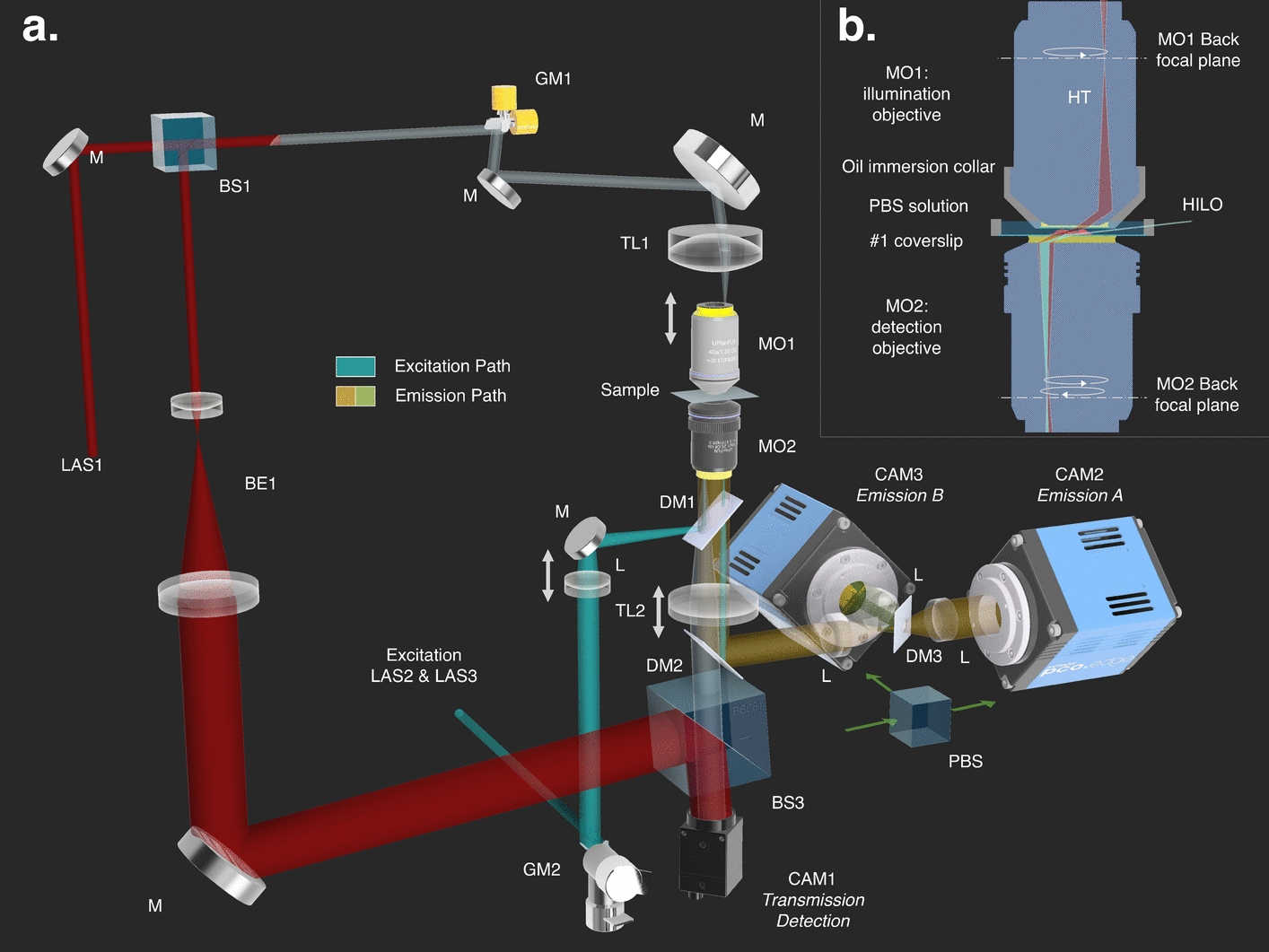
Multimodal HT system design. **a** A schematic of the HT system. L: achromatic doublet lens, M: mirror, BS: beam splitter, PBS: polarized beam splitter (optional), DM: dichroic mirror, MO: microscope objective lens, TL: tube lens, LAS: laser, GM: galvanometer mirror, BE: 10X beam expander, CAM: CMOS camera. Laser conditioning optics are omitted from the figure for simplicity. **b** Illustration of the coupling of the modalities in the system**.** HILO, high inclined laminated optical sheet, TIR, total internal reflection (for illustration only). A configuration with the oil-immersion illumination MO and collar is shown

Our compact telecentric DHM system integrates a two-axis galvanometer scanner to perform multi-angle illumination HT and a colocalized total internal reflection fluorescence (TIRF) module sharing a common detection microscope objective (MO) (Fig. 7). The HT modality probes and images the specimen with one wavelength for both tracking and tomographic RI reconstruction. The TIRF fluorescence module comprises two excitation lines and leverages two separate cameras for simultaneous two-color image acquisition.

The HT component of the system begins with a 660-nm laser (OBIS LX 660 nm, 100 mW, Coherent) that is split into object and reference arms with a non-polarized beam splitter (BS013, Thorlabs). The reference arm is further expanded with an achromatic beam expander (GBE10-A, Thorlabs) to approximate a planar reference wavefront prior to recombination. The object arm contains dual 4f relay subsystems (telecentric relays) that conjugate the galvanometer to the objective back focal plane (BFP) for angular beam steering at the sample. The illumination tilt angles are controlled via a two-axis galvanometer mirror (AT25-AT2412, DYUE). The steered beam is relayed through a 100 mm achromatic doublet to the BFP of the illumination condenser objective (MO1: UApo N340 40 × /1.35 oil or XLUMPLFLN 20 × /1.0 water, Olympus) as shown in Fig. 7b, producing variable incidence angles at the sample. The sample stage is translated in X and Y using encoded linear actuators (CMA-12CCCL, Newport) and a Z-axis stepper motor (LS-50, Applied Scientific Instruments). The transmission illumination passed through the sample to the detection objective (MO2: UApo N340 40 × /1.35 oil or PlanApo 60 × /1.45 oil, Olympus). After MO2, the transmitted holographic interference field is imaged by a 180 mm tube lens. The collimated object beam is then recombined with the reference at BS3 and is recorded by a CMOS camera (BFS-U3-20S4M-C, Teledyne Vision Solutions).

The fluorescence excitation path combines 561 nm (Compass 315 M, 25 mW, Coherent) and 488 nm (Sapphire 488-20, 20 mW, Coherent) laser lines dedicated to fluorescence excitation only, through a dichroic beam splitter. Another two-axis galvanometer mirror (AT25-AT2412, DYUE) performs the azimuthal scanning of the excitation beam on the detection objective BFP. The steered excitation beam then impinges upon a 125 mm focusing lens and passes through the excitation dichroic filter cube (52-852112-001 multiband dichroic, API; U-MF2 filter cube, Olympus) shared by the HT and fluorescence channels. The excitation spot on the BFP of MO2 is further adjusted by a motorized linear actuator (CMA-25CC, Newport) to switch between TIRF, HILO, and epifluorescence illumination. After separation from the DHM object beam downstream of TL2, the two-channel fluorescence emission is separated by a dichroic mirror (DM) (T647lpxr, Chroma Technologies) and directed to a pair of sCMOS camera (pco.edge 5.5, Excelitas) with 6.5 µm pixels, which is preceded by a telescope relay by doublets with focal lengths of 30 mm and 60 mm. A DM (T565lpxr, Chroma Technologies) or a polarized beam splitter (PBS251, Thorlabs) is inserted between the telescope for emission separation. Finally, the holographic telecentricity of the tube lens is adjusted using a linear actuator (CMA-12CC, Newport).

### Off-axis digital holography

Using a single illumination wavelength λ₁, the interference in off-axis digital holography by the collimated object ${O}_{1}$ and reference ${R}_{1}$ beams can be described by (Eq. 1):

1 $$I_{H} \left( {x,y} \right)\, = \,\left| {O_{2} } \right|^{2} \, + \,\left| {R_{1} } \right|^{2} \, + \,R_{1} \,^{ * } \,O_{1} \, + \,O_{1} \,^{ * } \,R_{1} ,$$

where ${I}_{H}$ is the distribution of the projected interference pattern intensity on the detector, and the sum of ${\left|{O}_{1}\right|}^{2}$ and ${\left|{R}_{1}\right|}^{2}$ representing the zero-order diffraction. The + 1 diffraction order, denoted as ${{R}_{1}}^{*}{O}_{1}$, arises from the interference of the reference wave with either the real or conjugate object wavefront, ${O}_{1}$, associated to propagation wave vectors ${k}_{obj}$. The resulting complex light field ${U}_{1}$, which encodes the interference information, can be filtered in the Fourier power spectrum by a circular filter mask. The phase map of the sample can be recovered as (Eq. 2):

2 $$\Phi_{1} \,\left( {x,y} \right)\, = \,\arctan \,\,\left[ {\frac{{{\mathrm{Im}} \left( {U_{1} \left( {x,y} \right)} \right)}}{{{\mathrm{Re}} \left( {U_{1} \left( {x,y} \right)} \right)}}} \right].$$

The phase map ${\Phi}_{1}\left(x, y\right)$ is initially wrapped between extrema $\pm \pi$, representing the OPD between the object beam and the reference beam. An unwrapping operation is then performed to recover the correct phase map in regions where phase variations exceeding the $\pm \pi$ range (Eq. 3):

3 $$OPD\left( {x,y} \right)\, = \,Unwrap\left( {\varphi_{1} \left( {x,y} \right)} \right)\,\frac{{\lambda_{1} }}{2\pi }\, = \,\left[ {{\rm N}_{1} \,\left( {x,y} \right)\, + \,\frac{{\varphi_{1} \,\left( {x,y} \right)}}{2\pi }} \right]\,\,\lambda_{1} ,$$

where OPD is a product of illumination wave propagation distance in the sample and the sample’s comprehensive RI, ${\varphi}_{1}$ denoting the wrapped phase, ${\mathbb{N}}_{1}$ as an integer multiplier for the full 2 $\pi$ range. The sample height or thickness, ${h}_{1}$, is computed, assuming uniform sample RI ${n}_{s}$ and medium RI ${n}_{m}$ along the optical path (Eq. 4):

4 $$h_{1} \,\left( {x,y} \right)\, = \,\frac{{OPD\,\left( {x,y} \right)}}{\Delta n},\,\,\Delta n\, = \,n_{s} \, - \,n_{m} .$$

### Particle tracking based on angular spectrum propagation and focus metrics

In the angular spectrum propagation model, the zero-order complex light field $U$ at the image plane $z=0$ can be expressed as in (Eq. 5):

5 $$U\left( {x,y,0} \right)\, = \,\int_{ - \infty }^{ + \infty } A \left( {\frac{\alpha }{\lambda },\frac{\beta }{\lambda },\,0} \right)\,\exp \,\left[ {i2\pi \left( {\frac{\alpha }{\lambda }\,x\, + \,\frac{\beta }{\lambda }\,y} \right)} \right]\,\,dxdy,$$

where $A\left(\frac{\alpha }{\uplambda },\frac{\beta }{\uplambda },0\right)$ is the propagation angle of the planar illumination. For each candidate reconstruction distance $z\in \mathrm{Z}$, we propagate $U\left(x,y,0\right)$ to obtain an axial image stack in Cartesian coordinates, as follows (Eq. 6):

6 $$U\,\left( {x,y,z} \right)\, = \,FFT^{ - 1} \,\left\{ {FFT} \right.\left\{ {U\left( {x,y,0} \right)} \right\}\, \cdot \,\exp \,\left. {\left( {iz\, \cdot \,k_{z} } \right)} \right\},$$

where the $z$ component of the wave vector ${k}_{obj}$ is ${k}_{z}= \sqrt{{k}^{2}-{{k}_{x}}^{2}-{{k}_{y}}^{2}}$. Among the reconstructed slices, we first localize a ROI around the particle of interest and improve contrast by subtracting time-averaged sample-free background. The focused slice, which is indicative of the particle axial localization, is identified by integrating the absolute values from the corresponding Fourier spectrum, which is logarithmically weighted to produce the sharpness metric ${F}_{FS}$ at the propagated plane $z$ (Eq. 7):

7 $$F_{FS} \, = \,\sum\nolimits_{x,y} {\log \left\{ {1\, + \,\left| {FFT\left\{ {U\left( {x,y,z} \right)} \right\}} \right|} \right.} \} .$$

The axial location of the tracked particle, ${z}_{Focused}$, is therefore indicated by the slice index with the highest integrated intensity value (Eq. 8):

8 $$z_{Focused} \, = \,\arg \max_{z \in Z} \left\{ {F_{FS} \left( z \right)} \right\}.$$

### Holotomographic reconstruction

**Fig. 8:**
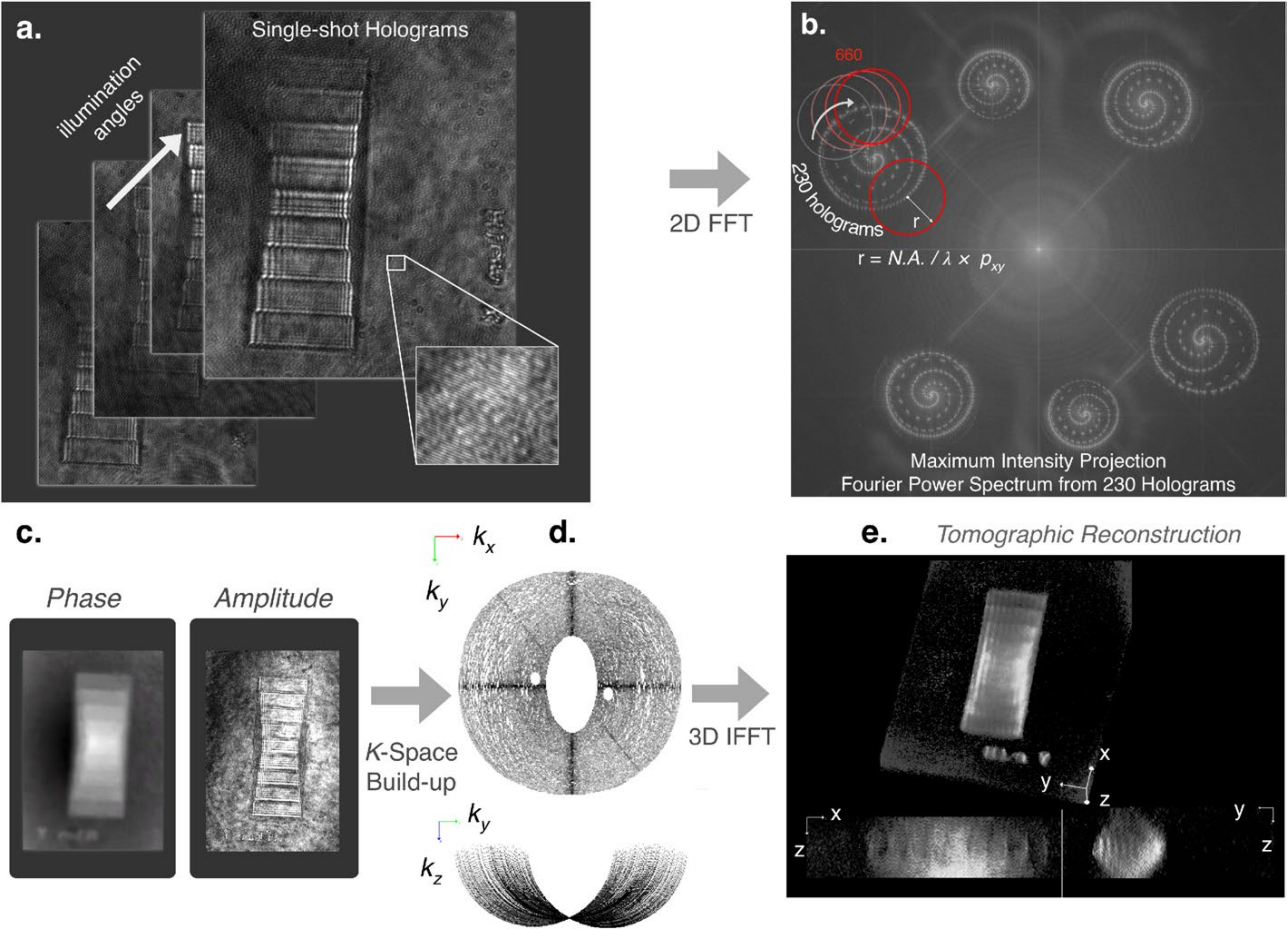
Field extraction and tomographic reconstruction pipeline of triple-wavelength HT. **a** Wavelength-multiplexed off-axis holograms are acquired by scanning the illumination angle (230 in total). A nano-printed step target sample is shown. Inset: Cropped image of raw hologram showing the + 1 order interference fringe. **b** Maximum-intensity projection of Fourier power spectra over all angles. Color-labeled loci mark the carrier positions. **c** Phase (left) and amplitude (right) retrieved for a nano-printed step target. **d** Angle-resolved complex fields are mapped to 3D k-space and inverted with an inverse Fourier transform. **e** The reconstructed refractive-index volume of the step target. Orthogonal slices of the tomogram are shown

We employed a tomographic reconstruction procedure similar to previously reported works [13, 15]. First, the sample’s complex optical fields were recovered from the spatially modulated interferograms $I_{H} \left( {x,y,\theta } \right)$ recorded under a set of illumination angles $\theta$ for the objective beam with k-vector ${k}_{obj\theta }$ (Fig. 8b–c). The unwrapped phase is then extracted using a circle filter in the Fourier power spectrum by the direction of the wave vector described in Eq. 2, forming an angle-encoded complex field $\left( {x,y;\theta } \right)\, = \,\left| U \right|\,\exp \,\left[ {i\angle U} \right]$, where *k*’s relation with the angles is given in Eq. 9:

9 $$k_{obj\theta } \, = \,\frac{2\pi }{\lambda }\,\left( {\sin \theta ,\,0,\cos \theta } \right).$$

Each Fourier coefficient $\tilde{U}_{obj\theta } \,\left( {k_{x} ,k_{y} } \right)$ associated with the complex fields is subsequently mapped into 3D k-space using the transformation, with each transverse frequency $\left( {k_{x} ,k_{y} } \right)$ having an axial component$k_{z}$:

$$\left( {k_{x} \, + \,k_{{obj,x^{,} }} \,k_{y} \, + \,k_{{obj,y^{,} }} \,k_{z} } \right),\, \cdot where \cdot k_{{z\left( {k_{x} ,k_{y} } \right)}} \, = \,\sqrt {k_{0}^{2} \, - \,k_{x}^{2} \, - \,k_{y}^{2} \,} ,$$

effectively centering the acquired 2D disk around the incident wavevector in 3D k-space using the Fourier diffraction theorem and inverted using the Rytov weak-scattering approximation [43]. These points are then sampled back into Cartesian space to obtain $\tilde{O}_{xyz} \left( {k_{x} ,k_{y} ,k_{z} } \right)$ which is the spectrum of the object’s scattering potential (Fig. 8d). Finally, the refractive-index tomogram $n\left( {x,y,z} \right)$ can be extracted by a 3D inverse Fourier transform (Eq. 10):

10 $$n\left( {x,y,z} \right)\, = \,n_{0} \sqrt {1\, - \,0\left( {x,y,z} \right)} .$$

Because the numerical apertures of the condenser and objective limit the collected scattering angles, the reconstruction suffers from an axial “missing cone” [15, 18]. We addressed this through iterative regularization with a non-negative constraint on the RI perturbations.

### Holographic and tomographic RI imaging

We optimized hologram interference + 1 order multiplexing with strategies previously reported by Jin et al*.* [44]: all detection cameras are rotated 45 degrees around the respective optical axes to facilitate higher interference fringe bandwidth and precise alignment across the modalities, whereas the illumination angle reference arm is optimized such that the interference pattern of the three wavelength are well separated.

The galvanometers are driven by a microcontroller (Arduino Nano, Arduino) which outputs analog position waveforms to modulate the illumination angles and issues hardware trigger pulses to the DHM camera after each mirror position to acquire the corresponding off-axis hologram. The galvanometer is set to a centered position during a single-shot particle tracking experiment. We use Pyhololab [45], a Python-based acquisition software for real-time QPI computation and tomographic reconstruction. The software communicates with the microcontroller to scan the illumination objective’s BFP across 230 angular positions, acquiring both a sample-free background series and subsequent sample series. During acquisition, phase retrieval of the angle-scanned holograms and reconstruction of the RI tomogram were performed on a workstation with a graphics processing unit (GPU) (RTX 3090, Nvidia). To aid visualization of the RI tomograms of the phantom and biological samples, cell masks generated by thresholding the DHM phase maps were applied to the tomogram.

During imaging, the brightfield camera exposure is set to 300 µs. At an acquisition rate of 50 frames per second (fps), a volumetric data set can be acquired in ~ 4 s. Processing of a 512 × 512 × 150-pixel volumetric tomogram required ~ 0.6 s on an RTX 3090 GPU, whereas a 1200 × 775 × 150 tomogram required 8.2 s.

**Fig. 9:**
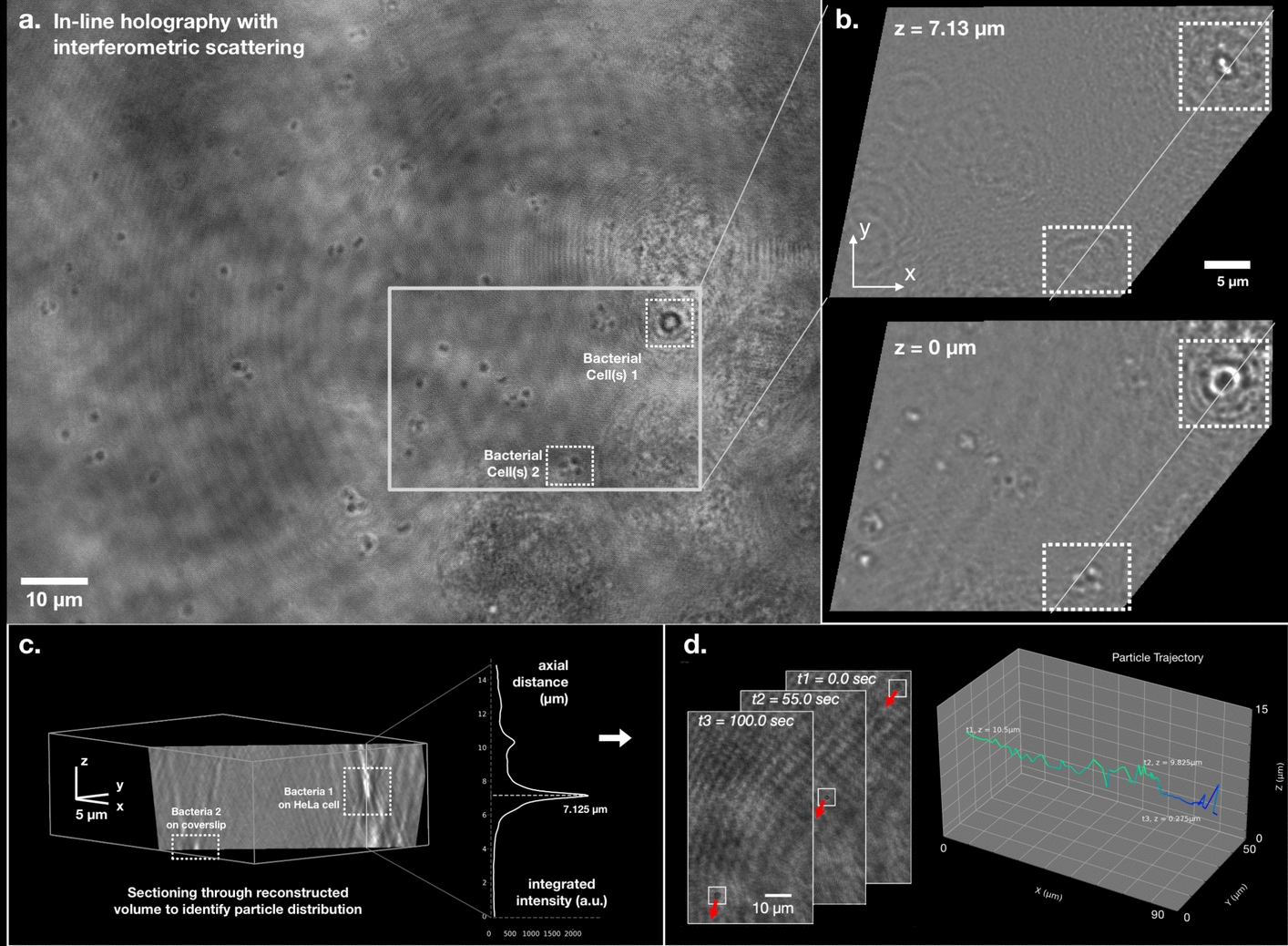
Procedure for spatial–temporal tracking of *N. gonorrhoeae* bacteria. **a** Colocalization of HeLa cells and *N. gonorrhoeae* bacteria is resolved by the DHM, where an ROI consists of bacteria on the glass coverslip and above the epithelial cell layer. **b** The numerically propagated ROI stack with background noise removed at reconstruction distance of 0 µm and 6.90 µm. **c** Volumetric rendering on the section taken in **b**. The axial location of the high-scattering bacteria cells can be recovered and visualized. **d** With a time-sequence, the 3D localizations of the particles can be linked together to form a spatial–temporal trajectory

For the pathogen tracking experiment (Fig. 9), image acquisition was performed at 5 fps. Numerical propagation was applied over a 15 µm reconstruction range with a step size of 0.1 µm (Fig. 9c). Bacterial lateral positions at each time point, as well as the temporal linking of 2D trajectories, were determined using trackpy [46]. Axial localization of each bacterium was obtained by numerical propagation combined with focus confirmation within a region of interest (ROI) centered around the bacterium (Fig. 9d). The overall processing speed for a 128 × 128 × 150 voxel ROI of a tracked particle at a given time point was 119.5 ms on an RTX 3090 GPU.

### System validation with nano-printed phantoms

To experimentally validate the multimodal HT system, two nanoscale additively manufactured phantoms were created: a set of step targets and a model of a fluorescently labeled vascularized lung organoid. Both phantoms were fabricated by two-photon polymerization (2PP), on a Nanoscribe Photonic Professional GT2 system (Nanoscribe GmbH, Karlsruhe, Germany) equipped with a femtosecond pulsed laser (λ = 780 nm, pulse width = 100 fs, repetition rate = 80 MHz).

The step targets (Fig. 1a**, **Fig. 8a) were fabricated with IP-Dip2 (Nanoscribe GmbH, Karlsruhe, Germany) resin that results in a refractive index of 1.547 after photopolymerization. The resin was deposited on a glass substrate and polymerized using a 63 × objective lens. The fabricated target consisted of five step-like structures, each with a lateral width of 15–25 μm and a height of 1 µm. As the sample does not contain a fluorescent label, we performed DHM and HT imaging with the step target immersed in distilled water (*n* = 1.33) during imaging.

The organoid phantom (Fig. 2a) was derived from the confocal laser scanning microscope (CLSM) image of a 94-week-old vascularized lung organoid. The sample was labeled by Cy5 and imaged on a CLSM (Nikon Eclipse Ti2-E) equipped with a 20 × objective lens (CFI Plan APO λ 20 × /0.75) using 640 nm excitation. The image stack was processed by removing small objects and connecting larger 3D morphologies. During additive manufacturing, fluorescent nanoparticles were incorporated into IP-Visio (Nanoscribe GmbH, Karlsruhe, Germany) photosensitive resin with a uniform RI of *n* = 1.52 post-polymerization. The nanoparticles, which were selected due to their low autofluorescence in the visible spectrum [47], were added at a concentration of 0.1 mg/mL. The structure was sectioned into stitched blocks of 285 × 285 µm and printed using a 25 × objective lens with laser power of 50 mW and a scan speed of 35 mm/s. After printing, the sample’s geometry and fluorescence properties were assessed on the same CLSM with 488 nm excitation wavelength and a 4 × microscope objective (Plan Fluor 4 × /0.13) (Fig. 2b). Subsequently, 1 µm diameter fluorescent polystyrene beads (Fluorospheres 580/605, ThermoFisher Scientific) with RI of *n* = 1.59 were added onto the top of the target by perfusion. Finally, the sample slide is covered by microscope immersion oil (MOIL-30, Olympus) with RI of *n* = 1.518 and sealed with a 0.15-mm-thick glass slide. The vascularized organoid was first imaged with bright field HT using oil immersion objective MO1 and MO2. For HILO fluorescence imaging, the sample is excited by the 488 nm laser, and had its emission spectra filtered by a 515/30 bandpass filter (HQ515—30 m, Chroma Technologies), while the fiducial fluorescent beads were excited by the 561 nm laser and a 594/45 filter (batch number 34-100615-000, Applied Precision Inc.).

### Live imaging of HeLa cell lines following exposure to *N. gonorrhoeae*

HeLa cells were maintained at 37 C/5% CO₂ in RPMI Medium 1640 with L-glutamine (Wisent Inc.) supplemented with 10% fetal bovine serum (FBS) and 1% GlutaMAX, then seeded on 27 mm glass-bottom dishes. Following our previous protocol [35], CEACAM1-4L-EYFP was generated in pEYFP-N1 and transiently expressed 24–48 h before assays. For the infection experiments, HeLa cells were seeded in a 27 mm Nunc glass base dish (Thermo Scientific) before transfection. *N. gonorrhoeae* strain MS11 expressing Opa_58_ [48] was grown on GC agar (Becton Dickinson) with Kellogg’s supplements and vancomycin, colistin, nystatin and trimethoprim (VCNT) (Becton Dickinson) at 37 °C with 5% CO₂ atmosphere overnight. The colony opacity (Opa) phenotype was verified by passaging under a dissecting microscope. Colonies were suspended in Dulbecco’s phosphate-buffered saline (D-PBS, with calcium and magnesium) (Wisent Inc.), pelleted and labeled with Texas Red-X-succinimidyl ester (Thermo Scientific) (10 µg mL⁻^1^, 1 h, RT, gentle rotation), and then washed four times in RPMI. Bacterial cells were inactivated by fixation with 4% paraformaldehyde and stored in PBS (Wisent Inc.) at 4 °C.

For both bacterial tracking and tomographic imaging, 50 µL of bacterial suspension was introduced under flow onto CEACAM1-EYFP HeLa monolayers. In the bacterial tracking experiments, live HeLa cells were first imaged prior to perfusion to obtain a reference free from bacteria-induced scattering. Following perfusion, the sample was held stationary for 5 min and then imaged continuously for 10 min to allow concurrent, single-shot HILO and bacterial tracking. For holotomography (HT) and volumetric HILO live imaging of the CEACAM response to bacterial infection, the sample is held stationary for an hour to allow for receptor recruitment before imaging. Fixed samples were also prepared to allow additional imaging and validation. After bacterial delivery by the same perfusion method, live HeLa cells were incubated for up to 3 h at 37 °C with 5% CO₂ to promote adhesion, followed by three PBS rinses to remove non-adherent bacteria. The samples were then fixed with 4% paraformaldehyde, washed, and stored in PBS at 4 °C until imaging.

### Multicolor HILO fluorescence imaging

The fluorescence microscopy component was first aligned to epifluorescence mode using the excitation stage. A sinusoidal pattern was then applied to the excitation galvanometer, steering it to reach a 12.5-degree divergence angle from the optical axis (Supplementary Information 4). The focused excitation spot would scan around the edge of the detection objective BFP to reach the supercritical condition, where the beam internally reflects near the coverslip region. The excitation angle control stage is then used to reposition the excitation angle to perform HILO sectioning. To acquire 3D fluorescence images in HILO mode, the sample stage was scanned axially. For biological samples, a 15-µm scan window with 0.25 µm or 0.5 µm step sizes were used, whereas synthetic targets were imaged over a 30 µm range with 1 µm steps. The sCMOS cameras were configured with a 1-s exposure time, capturing images in response to trigger signals from the acquisition program following each stage actuation.

For enhancing the image contrast and the spatial resolution of the fluorescence images, 3D Richardson–Lucy deconvolution was performed on the live cell images. Each fluorescence channel was deconvoluted using an averaged experimental point spread functions collected with 100 nm fluorescent polystyrene beads (Tetraspeck Microspheres, ThermoFisher Scientific) in distilled water. The resulting two-channel fluorescence images were then merged into a single combined fluorescence image, which can also be correlated with the independently measured 3D RI tomogram of the same sample.

### HILO anisotropy measurement of CEACAM1-EYFP

Fluorescence anisotropy imaging microscopy (FAIM) is used to spatially resolve association states of CEACAM between monomeric, dimeric and oligomeric states. To collect anisotropy emission of the CEACAM1-EYFP expressing HeLa monolayers, the DM separating the dual emission channel was replaced by the polarized beam splitter (PBS251, Thorlabs). Due to the optical path length difference induced by the beam splitter, we performed a quick realignment of the camera collecting the depolarized fluorescence, ${F}_{\perp }$, to match the parallel channel ${F}_{\parallel }$. The 515/30 bandpass filter was installed in front of the beam splitter. A 40 × oil objective (UApo N340 40 × /1.35, Olympus) was used for the experiment. A half waveplate (AQWP05M-600, Thorlabs) was placed in the path of the 488-nm laser and adjusted to output linearly polarized light. The excitation galvanometer was positioned in steady state at + 12 degrees to provide a minimal polarization angle from the coverslip. To correct for detector sensitivity differences, the G factor values was determined by calculating the intensity ratio of ${I}_{\parallel }$,the polarized fluorescence intensity parallel to the excitation upon ${I}_{\perp }$, the depolarized fluorescence intensity perpendicular to the excitation. G factor value calibration is performed in a 100 nM fluorescein standard solution which offers isotropic emission polarization across the two detectors, where an experimental value of 0.93 was determined. The anisotropy $r$ can be thus determined through the following equation (Eq. 11):

11 $$r\, = \,\frac{{I_{\parallel } \, - \,GI_{ \bot } }}{{I_{{\parallel \, + \,2GI_{ \bot } }} }}.$$

During post-processing, we first register the image stacks as described in Supplementary Information S2, before subtracting the images with the camera’s median background noise. We perform Axelrod’s high NA correction for our detection objective. Finally, cell masks are derived from thresholding the fluorescence images to derive the anisotropy measurements.

## Supplementary Information

Supplementary Material 1.

Supplementary Material 2.

## Data Availability

Data underlying the results presented in this paper are not publicly available at this time but may be obtained from the authors upon reasonable request.

## Abbreviations

CEACAM: Carcinoembryonic antigen‐related cell-adhesion molecule
DHM: Digital holographic microscopy
HT: Holotomography
HILO: High inclined laminated optical sheet

## References

[1] Gray-Owen SD, Blumberg RS. CEACAM1: contact-dependent control of immunity. Nat Rev Immunol. 2006;6:433–46. 10.1038/nri1864. 16724098 10.1038/nri1864

[2] Boulton IC, Gray-Owen SD. Neisserial binding to CEACAM1 arrests the activation and proliferation of CD4+ T lymphocytes. Nat Immunol. 2002;3:229–36. 10.1038/ni769. 11850628 10.1038/ni769

[3] Patel PC, Lee HSW, Ming AYK, Rath A, Deber CM, Yip CM, et al. Inside-out signaling promotes dynamic changes in the Carcinoembryonic Antigen-related Cellular Adhesion Molecule 1 (CEACAM1) oligomeric state to control its cell adhesion properties. J Biol Chem. 2013;288:29654–69. 10.1074/jbc.M113.504639. 24005674 10.1074/jbc.M113.504639 PMC3795263

[4] Sarantis H, Gray-Owen SD. The specific innate immune receptor CEACAM3 triggers neutrophil bactericidal activities via a Syk kinase-dependent pathway. Cell Microbiol. 2007;9:2167–80. 10.1111/j.1462-5822.2007.00947.x. 17506820 10.1111/j.1462-5822.2007.00947.x

[5] McCaw SE, Liao EH, Gray-Owen SD. Engulfment of *Neisseria gonorrhoeae* : revealing distinct processes of bacterial entry by individual Carcinoembryonic antigen-related cellular adhesion molecule family receptors. Infect Immun. 2004;72:2742–52. 10.1128/IAI.72.5.2742-2752.2004. 15102784 10.1128/IAI.72.5.2742-2752.2004 PMC387857

[6] Sadarangani M, Pollard AJ, Gray-Owen SD. Opa proteins and CEACAMs: pathways of immune engagement for pathogenic *Neisseria*. FEMS Microbiol Rev. 2011;35:498–514. 10.1111/j.1574-6976.2010.00260.x. 21204865 10.1111/j.1574-6976.2010.00260.x

[7] Werner LM, Palmer A, Smirnov A, Belcher Dufrisne M, Columbus L, Criss AK. Imaging flow cytometry analysis of CEACAM binding to Opa‐expressing *Neisseria gonorrhoeae*. Cytometry A. 2020;97:1081–9. 10.1002/cyto.a.24037. 32484607 10.1002/cyto.a.24037 PMC8062897

[8] Liu S-L, Li J, Zhang Z-L, Wang Z-G, Tian Z-Q, Wang G-P, et al. Fast and high-accuracy localization for three-dimensional single-particle tracking. Sci Rep. 2013;3:2462. 10.1038/srep02462. 23955270 10.1038/srep02462 PMC3746204

[9] Hsiao Y-T, Wu T-Y, Wu B-K, Chu S-W, Hsieh C-L. Spinning disk interferometric scattering confocal microscopy captures millisecond timescale dynamics of living cells. Opt Express. 2022;30:45233. 10.1364/OE.471935. 36522930 10.1364/OE.471935

[10] Tokunaga M, Imamoto N, Sakata-Sogawa K. Highly inclined thin illumination enables clear single-molecule imaging in cells. Nat Methods. 2008;5:159–61. 10.1038/nmeth1171. 18176568 10.1038/nmeth1171

[11] Chen X, Guo Q, Guan J, Zhang L, Jiang T, Xie L, et al. Single-molecule tracking in living microbial cells. Biophysics Reports. 2025;11:1. 10.52601/bpr.2024.240028. 40070662 10.52601/bpr.2024.240028 PMC11891077

[12] Goodman JW, Lawrence RW. Digital image formation from electronically detected holograms. Appl Phys Lett. 1967;11:77–9. 10.1063/1.1755043.

[13] Mann CJ, Yu L, Kim MK. Movies of cellular and sub-cellular motion by digital holographic microscopy. Biomed Eng OnLine. 2006;5:21. 10.1186/1475-925X-5-21. 16556319 10.1186/1475-925X-5-21 PMC1448199

[14] Shaked NT, Boppart SA, Wang LV, Popp J. Label-free biomedical optical imaging. Nat Photonics. 2023;17:1031–41. 10.1038/s41566-023-01299-6. 38523771 10.1038/s41566-023-01299-6 PMC10956740

[15] Balasubramani V, Kuś A, Tu H-Y, Cheng C-J, Baczewska M, Krauze W, et al. Holographic tomography: techniques and biomedical applications [Invited]. Appl Opt. 2021;60:B65. 10.1364/AO.416902. 33798138 10.1364/AO.416902

[16] Jin L, Yu Z, Au A, Serles P, Wang N, Lant JT, et al. P-TDHM: open-source portable telecentric digital holographic microscope. HardwareX. 2024;17:e00508. 10.1016/j.ohx.2024.e00508. 38327674 10.1016/j.ohx.2024.e00508 PMC10847153

[17] Horstmeyer R, Chung J, Ou X, Zheng G, Yang C. Diffraction tomography with Fourier ptychography. Optica. 2016;3:827. 10.1364/OPTICA.3.000827. 28736737 10.1364/OPTICA.3.000827 PMC5521281

[18] Kim G, Hugonnet H, Kim K, Lee J-H, Lee SS, Ha J, et al. Holotomography. Nat Rev Methods Primer. 2024;4:51. 10.1038/s43586-024-00327-1.

[19] Langehanenberg P, Ivanova L, Bernhardt I, Ketelhut S, Vollmer A, Dirksen D, et al. Automated three-dimensional tracking of living cells by digital holographic microscopy. J Biomed Opt. 2009;14:014018. 10.1117/1.308013. 19256706 10.1117/1.3080133

[20] Fonseca ESR, Fiadeiro PT, Pereira M, Pinheiro A. Comparative analysis of autofocus functions in digital in-line phase-shifting holography. Appl Opt. 2016;55:7663. 10.1364/AO.55.007663. 27661596 10.1364/AO.55.007663

[21] Wang A, Garmann RF, Manoharan VN. Tracking *E coli* runs and tumbles with scattering solutions and digital holographic microscopy. Opt Express. 2016;24:23719. 10.1364/OE.24.023719. 27828208 10.1364/OE.24.023719

[22] Mann CJ, Kim MK. Quantitative phase-contrast microscopy by angular spectrum digital holography. In: Conchello J-A, Cogswell CJ, Wilson T, editors. San Jose, CA; 2006 [cited 2025 Sept 28]. p. 60900B. 10.1117/12.645412

[23] Fan X, Healy JJ, Hennelly BM. Investigation of sparsity metrics for autofocusing in digital holographic microscopy. Opt Eng. 2017;56:1. 10.1117/1.OE.56.5.053112.

[24] Cuenat S, Andréoli L, André AN, Sandoz P, Laurent GJ, Couturier R, et al. Fast autofocusing using tiny transformer networks for digital holographic microscopy. Opt Express. 2022;30:24730. 10.1364/OE.458948. 36237020 10.1364/OE.458948

[25] Pavillon N, Benke A, Boss D, Moratal C, Kühn J, Jourdain P, et al. Cell morphology and intracellular ionic homeostasis explored with a multimodal approach combining epifluorescence and digital holographic microscopy. J Biophotonics Wiley. 2010;3:432–6. 10.1002/jbio.201000018. 10.1002/jbio.201000018 20306502

[26] Kim K, Park WS, Na S, Kim S, Kim T, Do Heo W, et al. Correlative three-dimensional fluorescence and refractive index tomography: bridging the gap between molecular specificity and quantitative bioimaging. Biomed Opt Express. 2017;8:5688. 10.1364/BOE.8.005688. 29296497 10.1364/BOE.8.005688 PMC5745112

[27] Shin S, Kim D, Kim K, Park Y. Super-resolution three-dimensional fluorescence and optical diffraction tomography of live cells using structured illumination generated by a digital micromirror device. Sci Rep. 2018. 10.1038/s41598-018-27399-w. 10.1038/s41598-018-27399-w PMC6004010 29907828

[28] Chowdhury S, Eldridge WJ, Wax A, Izatt JA. Structured illumination microscopy for dual-modality 3D sub-diffraction resolution fluorescence and refractive-index reconstruction. Biomed Opt Express. 2017;8:5776. 10.1364/BOE.8.005776. 29296504 10.1364/BOE.8.005776 PMC5745119

[29] Dong D, Huang X, Li L, Mao H, Mo Y, Zhang G, et al. Super-resolution fluorescence-assisted diffraction computational tomography reveals the three-dimensional landscape of the cellular organelle interactome. Light Sci Appl. 2020. 10.1038/s41377-020-0249-4. 10.1038/s41377-020-0249-4 PMC6987131 32025294

[30] Schürmann M, Cojoc G, Girardo S, Ulbricht E, Guck J, Müller P. Three-dimensional correlative single-cell imaging utilizing fluorescence and refractive index tomography. J Biophotonics. 2018;11:e201700145. 10.1002/jbio.201700145. 10.1002/jbio.201700145 28800386

[31] Xue Y, Ren D, Waller L. Three-dimensional bi-functional refractive index and fluorescence microscopy (BRIEF). Biomed Opt Express. 2022;13:5900. 10.1364/boe.456621. 36733730 10.1364/BOE.456621 PMC9872885

[32] Mattheyses AL, Shaw K, Axelrod D. Effective elimination of laser interference fringing in fluorescence microscopy by spinning azimuthal incidence angle. Microsc Res Tech. 2006;69:642–7. 10.1002/jemt.20334. 16770769 10.1002/jemt.20334

[33] Brunstein M, Teremetz M, Hérault K, Tourain C, Oheim M. Eliminating unwanted far-field excitation in objective-type TIRF. Part I. Identifying sources of nonevanescent excitation light. Biophys J. 2014;106:1020–32. 10.1016/j.bpj.2013.12.049. 24606927 10.1016/j.bpj.2013.12.049 PMC4026778

[34] Ellefsen KL, Dynes JL, Parker I. Spinning-spot shadowless TIRF microscopy. PLoS ONE. 2015;10:e0136055. 10.1371/journal.pone.0136055. 26308212 10.1371/journal.pone.0136055 PMC4550233

[35] Driouchi A, Gray-Owen SD, Yip CM. Correlated STORM-homoFRET imaging reveals highly heterogeneous membrane receptor structures. J Biol Chem. 2022;298:102448. 10.1016/j.jbc.2022.102448. 36063991 10.1016/j.jbc.2022.102448 PMC9539790

[36] Licata NA, Mohari B, Fuqua C, Setayeshgar S. Diffusion of Bacterial Cells in Porous Media. Biophys J. 2016;110:247–57. 10.1016/j.bpj.2015.09.035. 26745427 10.1016/j.bpj.2015.09.035 PMC4805881

[37] Bohrer CH, Xiao J. Complex Diffusion in Bacteria. In: Duménil G, Van Teeffelen S, editors. Phys Microbiol . Cham: Springer International Publishing; 2020 [cited 2025 Oct 2]. 15–43. 10.1007/978-3-030-46886-6_2

[38] Brown PT, Jabbarzadeh N, Meneses L, Swanson K, Pintuff A, Monakhova E, et al. Fourier synthesis optical diffraction tomography for kilohertz rate volumetric imaging. Sci Adv. 2025;11:eadr8004. 10.1126/sciadv.adr8004. 40802753 10.1126/sciadv.adr8004 PMC12346287

[39] Yang S, Kim J, Swartz ME, Eberhart JK, Chowdhury S. DMD and microlens array as a switchable module for illumination angle scanning in optical diffraction tomography. Biomed Opt Express. 2024;15:5932. 10.1364/BOE.535123. 39421770 10.1364/BOE.535123 PMC11482169

[40] Chen M, Liu H-Y, Ren D, Waller L. Multi-layered Born scattering model for 3D phase imaging with multiple scattering objects. Imaging Appl Opt 2018 3D AO AIO COSI DH LACSEA LSC MATH PcAOP . Orlando, Florida: OSA; 2018 [cited 2025 July 26]. p. CM3E.1. 10.1364/cosi.2018.cm3e.1

[41] Yang B, Lange M, Millett-Sikking A, Zhao X, Bragantini J, VijayKumar S, et al. DaXi—high-resolution, large imaging volume and multi-view single-objective light-sheet microscopy. Nat Methods. 2022;19:461–9. 10.1038/s41592-022-01417-2. 35314838 10.1038/s41592-022-01417-2 PMC9007742

[42] Driouchi A, Bretan M, Davis BJ, Heckert A, Seeger M, Silva MB, et al. Oblique line scan illumination enables expansive, accurate and sensitive single-protein measurements in solution and in living cells. Nat Methods. 2025;22:559–68. 10.1038/s41592-025-02594-6. 39966678 10.1038/s41592-025-02594-6 PMC11903300

[43] Rajan SD, Frisk GV. A comparison between the Born and Rytov approximations for the inverse backscattering problem. Geophysics. 1989;54:864–71. 10.1190/1.1442715.

[44] Jin L, Yu Z, Au A, Yip CM. Practical approach for optimizing off-axis telecentric digital holographic microscope design. Appl Opt. 2022;61:10490. 10.1364/AO.476308. 36607111 10.1364/AO.476308

[45] Yu Z, Jin L, Yip CM. DHM-viewer: comprehensive, open-source image processing suite for digital holographic microscopy. Biophys J. 2023;122:434a. 10.1016/j.bpj.2022.11.2348.

[46] Allan DB, Caswell T, Keim NC, van der Wel CM, Verweij RW. soft-matter/trackpy: v0.7. Zenodo; 2025 [cited 2025 Sept 28]. 10.5281/ZENODO.16089574

[47] Schmid M, Ludescher D, Giessen H. Optical properties of photoresists for femtosecond 3D printing: refractive index, extinction, luminescence-dose dependence, aging, heat treatment and comparison between 1-photon and 2-photon exposure. Opt Mater Express. 2019;9:4564. 10.1364/OME.9.004564.

[48] Gray‐Owen SD, Lorenzen DR, Haude A, Meyer TF, Dehio C. Differential Opa specificities for CD66 receptors influence tissue interactions and cellular response to *Neisseria gonorrhoeae*. Mol Microbiol. 1997;26:971–80. 10.1046/j.1365-2958.1997.6342006.x. 9426134 10.1046/j.1365-2958.1997.6342006.x

