# Supplementary material for: Multimodal holotomography for volumetric monitoring of host–pathogen interactions
Source: Biomed Eng Online. 2026 May 7;25:85. doi: 10.1186/s12938-026-01577-8 (PMC13317361; doi:10.1186/s12938-026-01577-8)
Supplement: Supplementary file 1 — Supplementary Material 1. [file 12938_2026_1577_MOESM1_ESM.docx]

**Supplementary Information for “Multimodal Holotomography for Volumetric Monitoring of Host-Pathogen Interactions”**

**Supplementary Information 1: Comparison to Existing multimodal Holotomography (HT) Systems**

| **Multimodal HT system** | **Method of forming HT images** | **Fluorescence Excitation** | **HT detection and Fluorescence Emission** | **Applicability for simultaneous multimodal imaging** | **Applicability for live imaging** |
| --- | --- | --- | --- | --- | --- |
| HT-X1 Plus,  Tomocube Life Sciences Inc. [1] | Non-interferometric deconvolution, axial sample scanning, DMD-modulated LED illumination | 4-channel LED epifluorescence or 4-line laser confocal spinning disk | Wide field or confocal detection | Yes | No (imaging speed is limited HT axial sample scanning) |
| Kim et al [2] | Off-axis Interferometric HT, DMD based | 3-channel LED epifluorescence | Single wide field detection | No (single detector shared among HT and fluorescence) | No |
| Shin et al [3] | Off-axis Interferometric HT, DMD based | Structured Illumination Super resolution | HT and fluorescence wide field detection | No (DMD shared between HT and SIM excitation) | No |
| Schürmann et al [4] | Wavefront detection phase imaging and sample rotation HT | Mercury lamp-based epifluorescence | HT and fluorescence wide field detection | Yes | No (imaging speed is limited by HT rotational sample scanning) |
| Chowdhury et al [5] | Common path Interferometric HT, SLM based | Multiplexed Structured Illumination Super resolution | HT and fluorescence wide field detection | Yes (Fourier order Multiplexing on SLM) | No (Imaging speed is limited on SLM angle scanning) |
| Dong et al [6] | Off-axis Interferometric HT, galvanometer based | Structured Illumination Super resolution HT and fluorescence | HT and fluorescence wide field detection | Yes (discrete HT and fluorescence beam path) | Yes (HT camera speed and fluorescence stage scanning limited) |
| Xue et al [7] | Non-interferometric, multi-slice propagation model | DMD-based Region of interest -selected excitation | Single wide field detection | No (requiring the preparation of fluorescence emitters below non-labeled sample) | Not suitable due to peculiar sample preparation technique |
| FS-ODT [8] | Off-axis Interferometric HT, DMD-based | Structured Illumination Super resolution | HT and fluorescence wide field detection | No (DMD shared between HT and SIM excitation) | Yes (up to 1 kHz for very sparse sample, lower for bulk cells and tissues) |
| Ours | Off-axis Interferometric HT, galvanometer based | HILO Excitation with azimuth and focal spot control | Simultaneous three channel detection (dual fluorescence channels) | Yes (discrete azimuth scanning for HT and HILO excitation; off-axis DHM mode; three channel detection) | Yes (HT camera speed and fluorescence stage scanning limited) |

**Supplementary Table S1: Comparison of Our HT implementation to existing multimodal HT systems.** LED, Light Emitting Diode; SIM, Structured Illumination Microscopy; DMD, Digital Micromirror Device; SLM, Spatial Light Modulator.

**Supplementary Information 2: Quantitative Resolution Metrics of the multimodal HT system**

To ensure the precision of our correlative measurements, we implemented a standardized alignment and calibration protocol using a target sample of mixed 500 nm and 100 nm fluorescent polystyrene beads (TetraSpeck Microspheres, ThermoFisher Scientific). The beads were mounted between two #1 coverslips and immersed in microscope immersion oil (MOIL-30, Olympus) ($n$ = 1.518). Fluorescence HILO imaging was performed using 488 nm excitation (emission wavelength $\approx515$ nm), while HT imaging utilized a 660 nm laser source. Data were acquired with a 6.5 µm pixel size sCMOS detector and a 1.45 NA microscope objective. The theoretical resolution of the modalities calculated based on the Abbe diffraction limit. The experimental resolutions were validated using full width half maximum (FWHM) measurement of diffraction limited PSF, and Fourier Ring Correlation (FRC) [9] on biological samples for real-world relevancy. These results are summarized in Table S2:

| **Modalities** | **Theoretical Lateral resolution (nm)** | **Theoretical Axial resolution (nm)** | **Measured Lateral FWHM resolution (nm)** | **Measured Axial FWHM resolution (nm)** |
| --- | --- | --- | --- | --- |
| HILO Fluorescence Microscopy | 178 | 744 | Raw: 514.7  with deconvolution:  331.8  Calibrated FRC on biological sample: ≥249.1 | Raw: 631.0  with deconvolution: 396.2 |
| HT Microscopy | 178 | 617 | 248.8  Calibrated FRC on biological sample: ≥251.9 | 637.8 |

**Supplementary Table S2.** Theoretical and Measured Resolution of HILO and HT Modalities

The results demonstrate that HT provides superior lateral resolution and structural morphology of the bacteria without the PSF distortion often induced by tissue fluorescence. However, HT is inherently limited by the missing-cone artifact, which results from the restricted range of illumination angles, leading to an elongation of the PSF in the axial (Z) direction. In contrast, HILO fluorescence provides specific molecular localization (CEACAM1-EYFP), and the implementation of 3D deconvolution allows us to overcome the out-of-focus blur typical of widefield techniques. The measured FWHM for deconvolved HILO ( $\approx332 nm$ lateral,  $\approx396 nm$ axial) provides the necessary precision to correlate CEACAM1 recruitment with the bacterial boundaries identified by HT. Inter-modality registration has also been demonstrated to achieve $\pm0.2 \mu m$ error for 100 nm beads samples (**Fig. S1a**), a metric we will further discuss in **Supplementary Information 4**. These results are demonstrated comparable to related works of fluorescence imaging and HT.


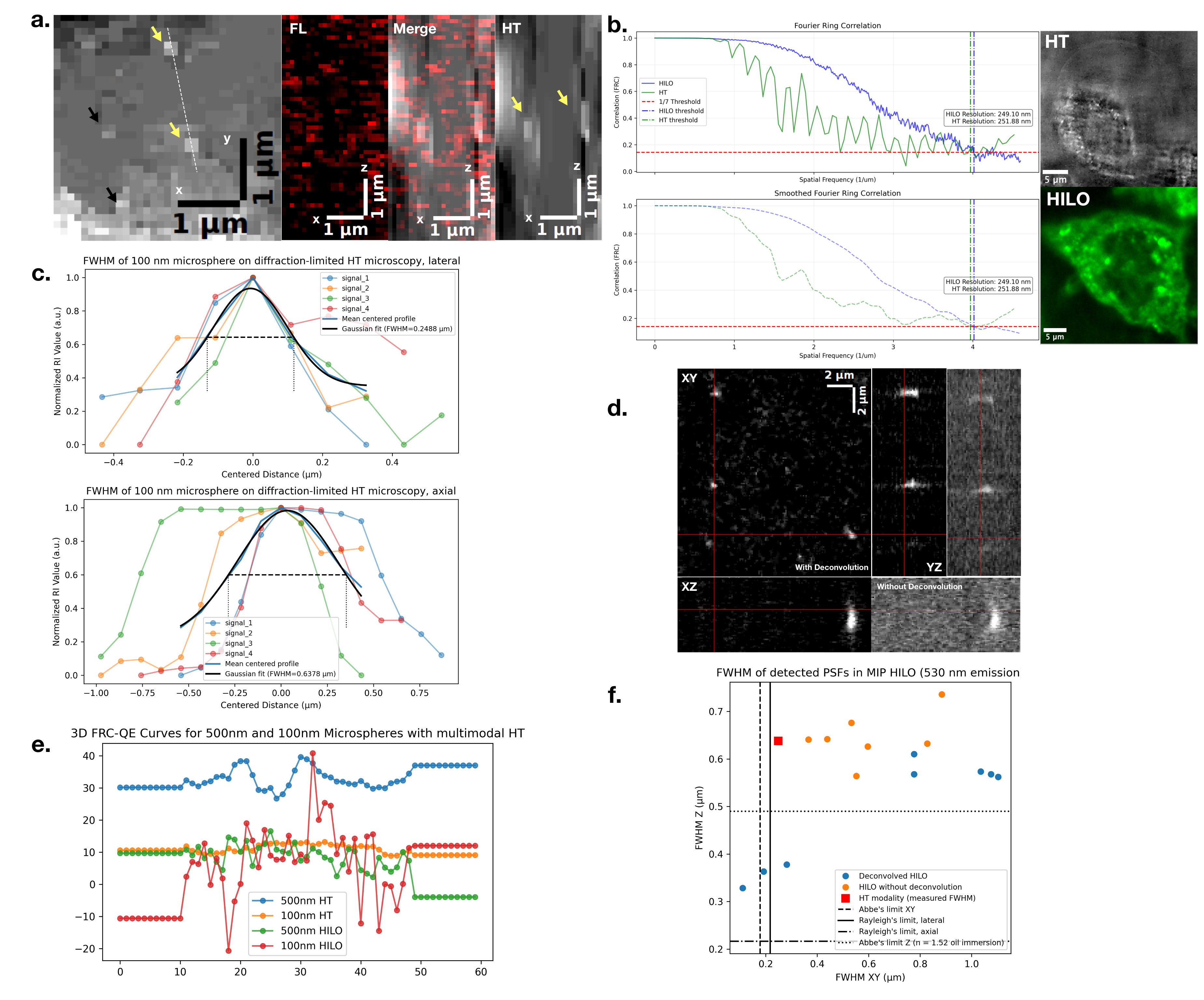
**Supplementary Figure S1:** Validation of HILO Fluorescence and HT microscopy resolution with 500 nm and 100 nm fluorescent polystyrene beads. **a** Representative *en face* (XY) HT refractive index (RI) contrast image of four 100 nm microspheres (yellow arrows). To the right, X-Z re-slices show the corresponding HT and HILO fluorescence (FL) line profiles, demonstrating the spatial correlation between RI and fluorescence signals. **b** FRC results for respective biological images of the modalities. left: FRC curves with fixed 1/7 threshold for HILO (blue) and HT (green); right: representative images (grey: HT, green: HILO: CEACAM1-EYFP) from which the FRC were derived. c Quantitative FWHM analysis for the HT modality. (Top) Lateral line profiles of four 100 nm microspheres with a mean Gaussian fit yielding a FWHM of 248.8 nm. (Bottom) Axial line profiles yielding a mean FWHM of 637.8 nm. **d** Visual comparison of HILO fluorescence PSFs in XY, YZ, and XZ planes. The application of Richardson-Lucy deconvolution significantly reduces the low intensity lobes associated with the widefield point spread function, effectively sharpening the axial localization. **e** 3D Fourier Ring Correlation (FRC) - Quality Estimation (QE) [10] curves for 500 nm and 100 nm microspheres across both modalities. Analysis indicates that while HILO resolution may be shot-noise limited when imaging 100 nm samples, HT provides consistently high-quality scores due to its label-free, high-contrast brightfield illumination. f Summary scatter plot of measured FWHM (Z vs. XY) for all modalities. HT (red square) is notably limited by the missing frequency artifact, which constrains its axial resolution compared to its superior lateral performance. HILO data points (blue and orange) illustrate the resolution enhancement achieved through deconvolution (blue), moving the system closer to the theoretical Abbe and Rayleigh limits.

**Supplementary Information 3: Analysis of HT Reconstruction Error in High-Refractive Index Mismatch and Scattering Approximations**

The fidelity of refractive index (RI) tomograms in HT is highly sensitive to the RI contrast (Δn) between the sample and the immersion medium. Standard HT reconstruction algorithms generally operate under the Rytov or Born approximations, which assume "weak scattering" conditions. When the RI mismatch is high, multiple scattering events and significant phase delays occur, violating these linear assumptions and introducing systematic errors into the reconstructed tomogram. To quantify these effects, we performed a benchmark analysis using standardized, non-fluorescent 4.5 µm polystyrene microspheres ( $n$ = 1.59). We compared two experimental conditions: *High Mismatch*: Beads immersed in distilled water ($n$ = 1.33), and *Low Mismatch*: Beads mounted in microscope immersion oil (MOIL-30, Olympus) ($n$ = 1.518). As shown in **Fig. S2a**, high RI contrast in the water-immersed samples leads to prominent "halo" and "squeezing" artifacts. The reconstructed XZ sections exhibit an axially stretched, ellipsoidal morphology rather than the true spherical shape. Mid-line profiles indicate that the recovered RI significantly underestimates the ground truth within the bead's interior while overestimating it in the surrounding "halo" regions. Conversely, when the RI is better matched using immersion oil (**Fig. S2b**), the reconstructed tomogram demonstrates superior morphological accuracy and sphericity. The mid-line RI profiles align more closely with the ideal top-hat distribution, although a slight underestimation remains due to the "missing cone" of information inherent in limited-angle tomography. We quantified these errors using the Root Mean Square Error (RMSE) of the RI values across all Z-planes (Fig. S2c). In the water-immersed samples, the RMSE spikes significantly within the bead volume, reaching peaks above 0.10 RI units. In oil-immersed samples, the error remains relatively low and stable. Aggregated data (Fig. S2d) show that the mean RMSE for water-immersed beads (≈0.061) is nearly triple that of the oil-immersed beads (≈0.023).

While we did not achieve perfect RI matching (<1% mismatch) in this study, these results underscore the importance of utilizing biocompatible RI-matching media to minimize reconstruction artifacts, which is a strategy that has been shown to significantly improve accuracy in live-cell imaging contexts [11] such as for nematode imaging. Additionally, regularizations strategies has been validated to be instrumental [11] in achieving improved accuracy for 3D RI reconstruction.


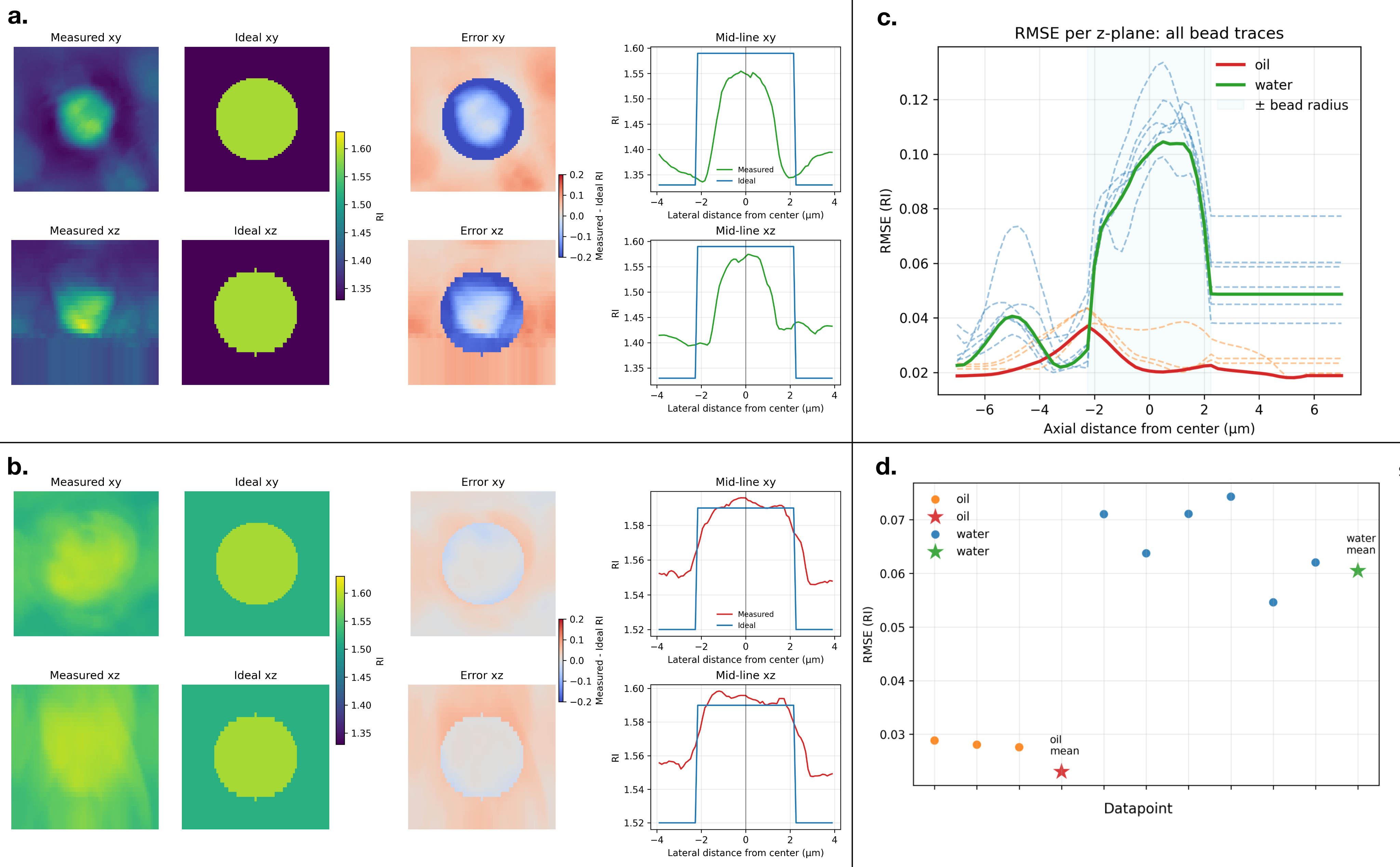


**Supplementary Figure S2.** Quantification of HT reconstruction errors under varying RI-mismatch conditions. **a** Reconstruction of a 4.5 µm polystyrene bead in water ($n$ = 1.33). Panels show measured vs. ideal RI maps in XY and XZ planes, spatial error maps, and mid-line RI profiles. Significant axial distortion and "halo" artifacts are visible. **b** Corresponding reconstruction of a bead in immersion oil ($n$ = 1.518). Reduced Δn leads to improved sphericity and more accurate RI recovery. **c** RMSE of the RI as a function of axial distance from the bead center for all traces ($n$ = 1.33) Red lines: oil; Green lines: water. The shaded region represents the bead radius. **d** Comparison of total mean RMSE between oil and water immersion, where the RMSE for the oil and water mean are derived from averaging the tomograms from the individual bead morphologies. The mean RMSE for water ($RMSE\approx0.061$, green star) is significantly higher than for oil ( $RMSE\approx0.023$, red star), highlighting the impact of the weak scattering approximation violation.

**Supplementary Information 4: Registration Accuracy Between Imaging Modalities**

We use the following alignment protocol at the beginning of an experiment: a white LED illumination source (spectral range from 450 nm to 690 nm) is set up in transmission over a target sample comprises of 500 nm diameter fluorescent polystyrene beads (Tetraspeck Microspheres, ThermoFisher Scientific) mounted between two #1 coverslips and immersed in distilled water ($n$ = 1.33). A non-polarized beam splitter (BS013, Thorlabs) is installed to split the transmission image between the two detectors. The cameras in the system are set to simultaneously acquire a stack image with 15 µm range and 0.25 µm step sizes, performed with the z-axis sample stage. The resultant bright field images are registered in ImageJ using the Registration Plugin (Linear Stack Alignment with SIFT, Affine transformation, with interpolation) or a custom Python script implementing Enhanced Correlation Coefficient registration in Pyhololab, which performs registration during live imaging. Due to the mismatched detector sizes and pixel sizes between the DHM camera and the fluorescence cameras, we crop the fluorescence images to match the Holotomography ROI slices; the second fluorescence channel is registered onto the first, followed by the DHM camera. The resultant affine transformation serves as the initial transformation estimation for the subsequent image post-processing of the given experiment, during which the process is performed again using the same protocol. It is noted that the initial transformation is derived essentially from a multicolor dataset; however, due to the use of achromatic optics in the system and the subsequent re-registration, we demonstrate the resultant registration accuracy was sufficient for correlated imaging. Previously in Supplementary Information S1, we have also established a sufficient cross-modality accuracy of same-sensor brightfield-to-fluorescence registration accuracy (≤ measured resolution limit of each modality type), thus this section will focus on discussing intra-modality detector matching to improve interpretability of our registration results.

We validated registration accuracy through qualitative landmark tracking and quantitative phase correlation analysis (Supplementary Fig. S3). For qualitative validation (3D Landmarks), 500 nm beads were selected as landmarks. 3D binary blob morphologies were segmented using manual selected seeding points, from which the center of mass for each bead is computed. The Euclidean distance between corresponding bead centers in registered channels was calculated (**Fig. S3b**). For quantitative validation, channel-wise standard deviation (STD) projections [12] and phase cross-correlation were used. The detected peak in the cross-correlation map indicates the residual translation error between the "Reference" and "Moving" channels (**Fig. S3d**). Visual inspection of the XY overlay (Fig. S3a) and XZ axial sections (Fig. S3c) demonstrates a significant reduction of mismatch post-registration. Specifically, axial validation shows that the combined pre-registration offset of 2.592 µm was successfully corrected, resulting in a singular interferometric PSF overlapping profiles of the PSF in the STD midline projection. We would like to note, however, that the uncompounded axial offset cannot be further differentiated on a pixel level in the standard deviation projection. In practice, the detectors should be always axially calibrated before imaging to ensure that the affine transformation is effective across the z-stack of images.

Quantitative results are summarized in Table S3. The lateral cross correlation error was reduced from ~1.09 µm to 0.216 µm, while the mean 3D Euclidean displacement between landmarks was halved, reaching sub-micron precision (≤0.50 µm).

| **Error Types** | **Phase cross-correlation offset (µm)** | **Qualitative landmark offset (µm)** |
| --- | --- | --- |
| Overall Euclidian error, before registration | 1.087 | 1.03 |
| Overall Euclidian error, after registration | 0.216 | ≤0.5 |

**Supplementary Table S2.** Cross-modality Error of HILO and HT Modalities before and after registration


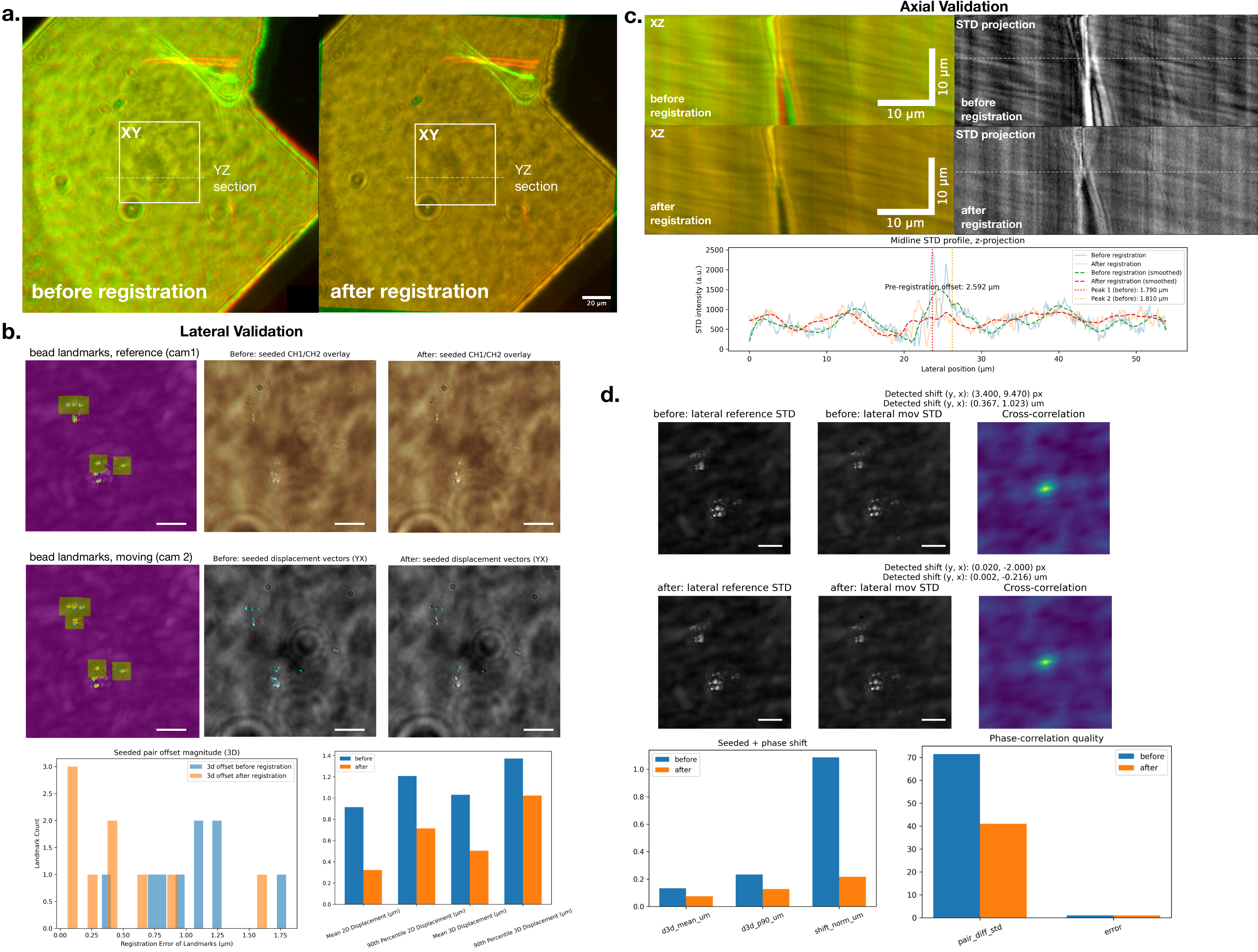


**Supplementary Figure S3: Multi-modal Registration Accuracy. a** Widefield overlay of HT and HILO channels before and after registration (XY plane). Colormap overlays: detector 1: red; detector 2: green. Qualitatively, a more “yellow” composite image represents a higher degree of registration. Scale bar: 20 µm. **b** Lateral validation. (Top) Landmark identification and quiver plots in reference and moving cameras using manually selected seeds. (Bottom Left) Histogram of 3D offset magnitudes showing a shift toward the 0–0.25 µm bin post-registration. (Bottom Right) Reduction in 2D and 3D displacement metrics. **c** Axial Validation. XZ sections and STD projections show the correction of a 2.592 µm combined Euclidean offset of the interferometric PSF. After affine registration, the uncompounded axial offset cannot be further differentiated on a pixel level in the std projection. Midline profiles confirm the alignment of peaks post-registration. **d** Phase cross-correlation Analysis. Cross-correlation maps show the sharpening of the correlation peak. Bar charts indicate the "before" (blue) and "after" (orange) detected shifts, confirming a final lateral error of 0.216 µm. Scale bars for **b, c, d**: 10 µm.

**Supplementary Information 5: Assessment of Azimuth-scanning HILO excitation**

Azimuth-scanning HILO excitation was implemented by a combination of the linear adjustment stage and a two-axis galvanometer system. Like the scanning HT system, the excitation galvanometer is driven by a microcontroller (Arduino Nano, Arduino) connected to two 12-bit Digital to Analog Converters (DAC) (MCP4921, Microchip Technology) through a custom circuit. The DAC translated 4096 discrete angular steps outputs voltages from 0 to 4.096 volt with a ±0.02 volt offset error and 4.5 µs settling time, translating to ± 25-degree galvo mirror displacement. The galvo’s angular repeatability was verified by the DHM Fourier power spectrum to be ±0.31 degrees. For beam-homogenized HILO usage, the galvo received a sinusoidal input with a 100 µs delay between each scan point. A full back focal plane (BFP) scan is performed with 80 positions, providing ≥100 scans during the camera integration time (1 second for CEACAM1-EYFP acquisition).

We verified the performance of azimuth scanning through both qualitative and quantitative means. A 40× objective was used in this experiment to capture additional field of view; the cameras acquired with 2×2 binning to eliminate discrepancies from detector oversampling. With the same input diameter of the excitation beam, azimuth scanning provides 65.1% larger excitable field of view and covers 23.2% HeLa cells and 30.9% more counts of N. gonorrhoeae bacteria respectively. We further conducted spectral signal to noise ratio analysis to measure the impact of azimuthal scanning on laser speckle noise and interference fringes removal with the following equation, where $P_{signal}$ and $P_{noise}$are mean intensity of the pixels in the power spectrum containing the pattern and the power spectrum of the image without the pattern, respectively:

$${SNR}_{db}=10 \cdot{log}_{10}\left( \frac{P_{signal}}{P_{noise}} \right), (1)$$

The Noise index is subsequently calculated by spectrum subtraction and computing the root mean square ratio of the difference image:

$${Noise}_{db}=20 \cdot{log}_{10}\left( \frac{{RMS(FFT}_{diff})}{{RMS(FFT}_{signal})} \right), (2)$$

With azimuth scanning, noise structure power was reduced by 14.43 dB (Supplementary Figure S4b). Fourier Ring Correlation also corroborate the findings and demonstrate a higher spatial resolution for the azimuth-scanned image.


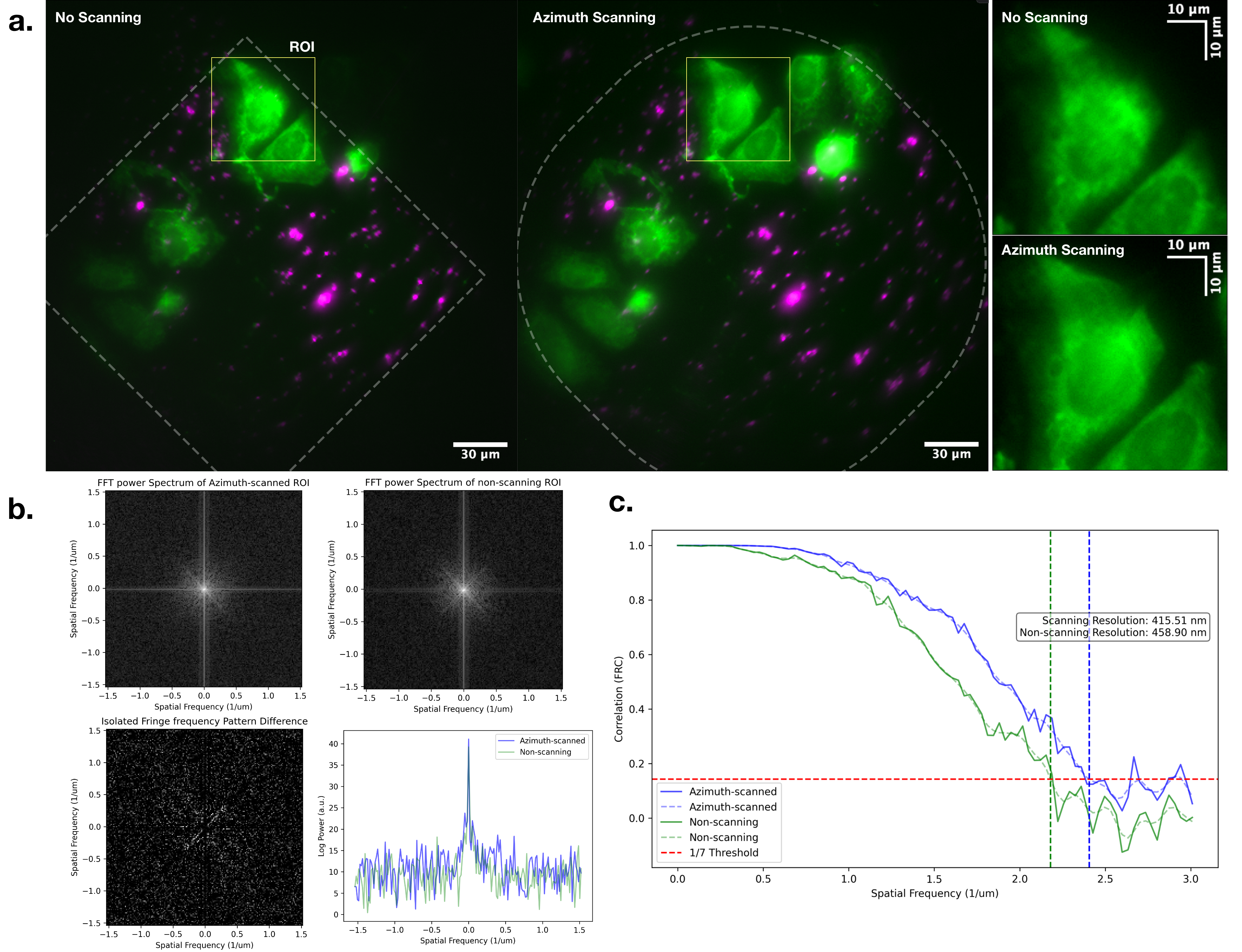


**Supplementary Figure S4: Qualitative and quantitative comparison of two types of HILO excitatio**n. **a** Qualitative field of view and IQ comparison. left: Two color HILO image with no beam scanning (Green: CEACAM1 (EYFP); Magenta: *N. gonorrhoeae* Opa58 (Texas Red); middle: HILO image with azimuth scanning. The dashed grey line illustrates the area where the fluorescence label has been sufficiently excited; right top: ROI from HILO image with no scanning; right bottom; ROI with azimuth scanning. **b** spectral signal to noise ratio analysis of the HILO ROIs. Top left: log scaled FFT power spectrum of azimuth-scanned ROI. Top right: power spectrum of the non-scanned ROI; bottom left: Isolated low frequency fringe noise present in the non-scanned ROI; bottom right: spectrum power comparison between the two ROIs by taking the midline. **c** Fourier Ring Correlation (FRC) analysis of the two ROIs. The Azimuth-scanned ROI demonstrates a higher spatial resolution. Deconvolution was not applied in these images.

**Bibliography**

1. Lee MJ, Lee J, Ha J, Kim G, Kim H-J, Lee S, et al. Long-term three-dimensional high-resolution imaging of live unlabeled small intestinal organoids via low-coherence holotomography. Exp Mol Med. 2024;56:2162–70. https://doi.org/10.1038/s12276-024-01312-0

2. Kim K, Park WS, Na S, Kim S, Kim T, Do Heo W, et al. Correlative three-dimensional fluorescence and refractive index tomography: bridging the gap between molecular specificity and quantitative bioimaging. Biomed Opt Express. 2017;8:5688. https://doi.org/10.1364/BOE.8.005688

3. Shin S, Kim D, Kim K, Park Y. Super-resolution three-dimensional fluorescence and optical diffraction tomography of live cells using structured illumination generated by a digital micromirror device. Sci Rep [Internet]. Springer Science and Business Media LLC; 2018 [cited 2025 July 26];8. https://doi.org/10.1038/s41598-018-27399-w

4. Schürmann M, Cojoc G, Girardo S, Ulbricht E, Guck J, Müller P. Three‐dimensional correlative single‐cell imaging utilizing fluorescence and refractive index tomography. J Biophotonics. 2018;11:e201700145. https://doi.org/10.1002/jbio.201700145

5. Chowdhury S, Eldridge WJ, Wax A, Izatt JA. Structured illumination microscopy for dual-modality 3D sub-diffraction resolution fluorescence and refractive-index reconstruction. Biomed Opt Express. 2017;8:5776. https://doi.org/10.1364/BOE.8.005776

6. Dong D, Huang X, Li L, Mao H, Mo Y, Zhang G, et al. Super-resolution fluorescence-assisted diffraction computational tomography reveals the three-dimensional landscape of the cellular organelle interactome. Light Sci Appl [Internet]. Springer Science and Business Media LLC; 2020 [cited 2025 July 26];9. https://doi.org/10.1038/s41377-020-0249-4

7. Xue Y, Ren D, Waller L. Three-dimensional bi-functional refractive index and fluorescence microscopy (BRIEF). Biomed Opt Express. Optica Publishing Group; 2022;13:5900. https://doi.org/10.1364/boe.456621

8. Brown PT, Jabbarzadeh N, Meneses L, Swanson K, Pintuff A, Monakhova E, et al. Fourier synthesis optical diffraction tomography for kilohertz rate volumetric imaging. Sci Adv. 2025;11:eadr8004. https://doi.org/10.1126/sciadv.adr8004

9. Nieuwenhuizen RPJ, Lidke KA, Bates M, Puig DL, Grünwald D, Stallinga S, et al. Measuring image resolution in optical nanoscopy. Nat Methods. 2013;10:557–62. https://doi.org/10.1038/nmeth.2448

10. Preusser F, Dos Santos N, Contzen J, Stachelscheid H, Costa ÉT, Mergenthaler P, et al. FRC-QE: a robust and comparable 3D microscopy image quality metric for cleared organoids. Robinson P, editor. Bioinformatics. 2021;37:3088–90. https://doi.org/10.1093/bioinformatics/btab160

11. Lee D, Lee M, Kwak H, Kim YS, Shim J, Jung JH, et al. High-fidelity optical diffraction tomography of live organisms using iodixanol refractive index matching. Biomed Opt Express. 2022;13:6404. https://doi.org/10.1364/BOE.465066

12. Cano-García AE, Lázaro JL, Infante A, Fernández P, Pompa-Chacón Y, Espinoza F. Using the Standard Deviation of a Region of Interest in an Image to Estimate Camera to Emitter Distance. Sensors. 2012;12:5687–704. https://doi.org/10.3390/s120505687

**Supplementary Data**

**
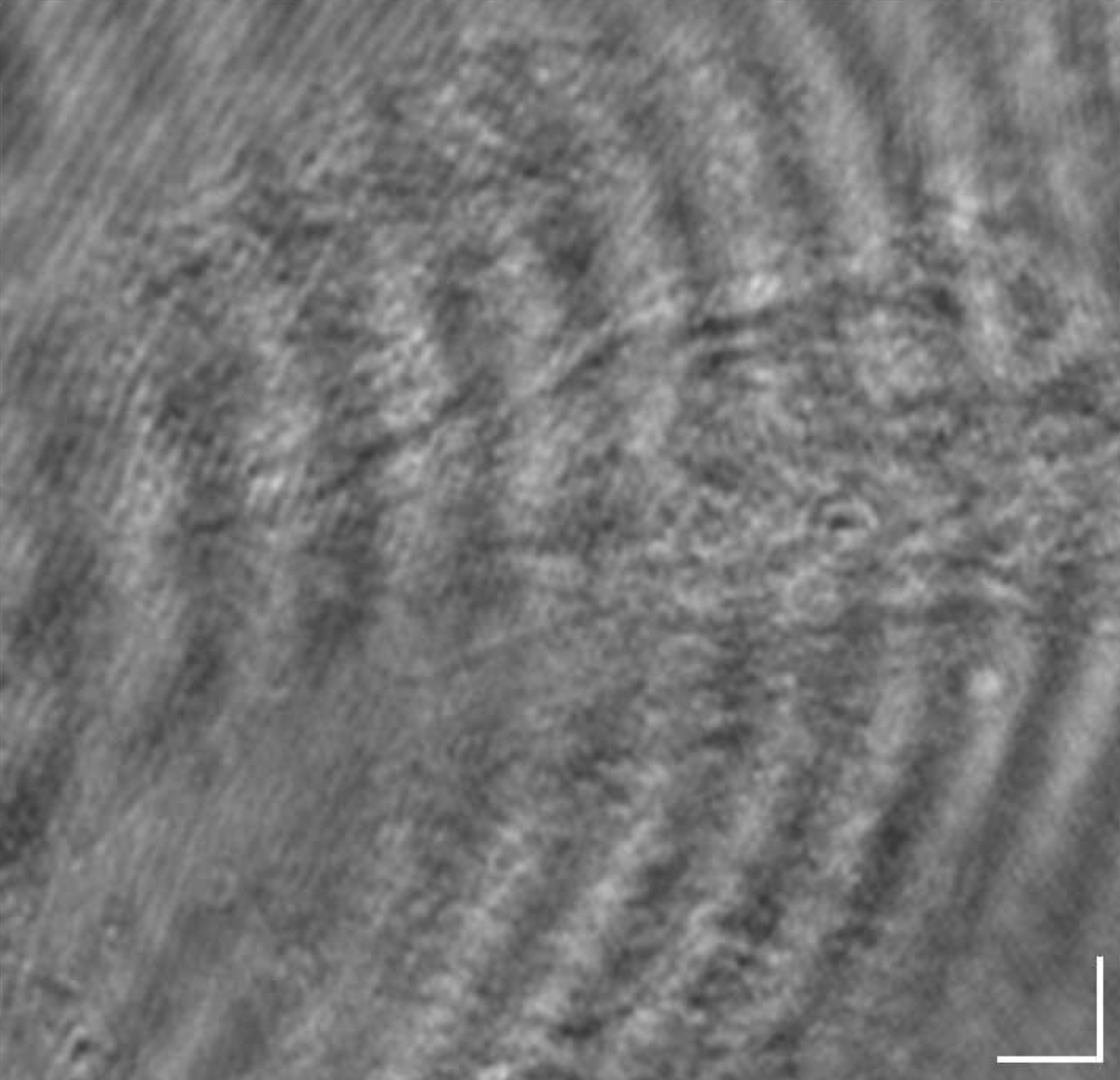
**

**Video S1: inline DHM video of *Neisseria Gonorrhoeae* bacteria being flowed over HeLa cell monolayer. Scale bar: 5 µm**
